# Site-specific azide-acetyllysine photochemistry on epigenetic readers for interactome profiling†
†Electronic supplementary information (ESI) available: Full experimental details, synthesis and characterizations of compounds, supplementary data, figures and references. See DOI: 10.1039/c7sc00284j



**DOI:** 10.1039/c7sc00284j

**Published:** 2017-03-14

**Authors:** Babu Sudhamalla, Debasis Dey, Megan Breski, Tiffany Nguyen, Kabirul Islam

**Affiliations:** a Department of Chemistry , University of Pittsburgh , Pennsylvania 15260 , USA . Email: kai27@pitt.edu

## Abstract

Chemical modifications on DNA, RNA and histones are recognized by an array of ‘reader’ modules to regulate transcriptional programming and cell fate. However, identification of reader-specific interacting partners in a dynamic cellular environment remains a significant challenge. Herein, we report a chemoproteomic approach termed ‘interaction-based protein profiling’ (IBPP) to characterize novel interacting partners of potentially any reader protein. IBPP harnesses a photosensitive amino acid introduced into the hydrophobic pocket of a reader module to crosslink and enrich transient interacting partners that are inaccessible to traditional methods. Using bromodomain-containing protein 4 (BRD4) as a paradigm, we engineer an ‘aromatic cage’ of the bromodomain to introduce 4-azido-l-phenylalanine (*p*AzF) without compromising its ability to recognize acetylated lysine residues in histone proteins. We establish the binding efficiency, substrate specificity and crosslinking ability of the engineered ‘reader’ module in biochemical assays. Applying IBPP, we uncovered novel acetylated interacting partners of BRD4, such as transcription factors, expanding on its previously unappreciated role in diverse biological processes. By setting up an azide-acetyllysine photoreaction deep inside the bromodomain aromatic cage as a means to detect protein acetylation, our approach provides a potentially general platform for rapid and unbiased profiling of interacting partners of diverse epigenetic readers whose functions in eukaryotic gene regulation remain convoluted.

## Abstract 2

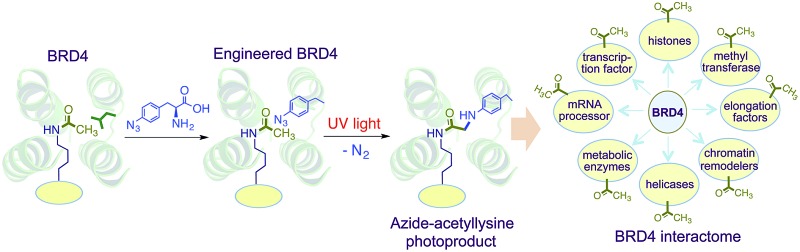
The hydrophobic pocket of the epigenetic reader protein BRD4 has been engineered to carry a photosensitive amino acid to identify novel interacting partners, providing mechanistic insights into BRD4’s function in transcription and beyond.

## Introduction

Post-synthetic modifications (PSMs) such as methylation in DNA and acetylation in histones contribute significantly to the heritable changes in gene expression essential for cellular differentiation and lineage commitment.^1^ The biological functions of dynamic PSMs are primarily mediated by the conserved ‘reader’ proteins that recognize these ‘marks’ to initiate downstream signaling *via* their interacting partners.^2^ The human genome encodes ∼500 such readers with evolutionarily conserved domains, such as the bromodomain and the plant homeodomain (PHD) that specifically bind acetylated and methylated histones, respectively.^3^ Recent proteomic studies have identified essential non-histone proteins (*e.g.* transcription factors) that carry PSMs and can potentially be recognized by the same set of readers.^4^ How such a large ensemble of readers, either alone or in combination, function in chromatin and non-chromatin contexts is largely unknown, primarily due to the lack of exhaustive profiling data of their distinct interactome.

The biological consequence of reader-mediated recognition of PSMs is highly dynamic and context dependent. For example, binding of H3K4Me_3_ by the TAF3-PHD of transcription complex TFIID results in gene activation; however, recognition of the same mark by ING2-PHD, a subunit of the histone deacetylase complex, leads to rapid gene repression.^2^ Combinatorial recognition of methylation and acetylation marks on the same nucleosome by the tandem bromodomain and PHD of transcription factor BPTF further exemplifies the complex nature of reader-modification interactions in nucleosome-templated processes.^5^ Although novel technologies have been developed to identify readers, a method for unbiased profiling of interacting partners of a specific reader is lacking. Available methods are either based on *in vitro* binding of modified truncated peptides to recombinant readers or the use of cell lysates as a potential source of ‘effector’ molecules.^6–8^ Such peptide-based assays are not suitable for fully recapitulating the highly dynamic interactions between modified proteins and their readers.^2^ This becomes particularly challenging in the presence of other gene regulatory complexes in the chromatin landscape where both DNA and histone modifications regulate reader functions. Traditional enrichment methods can identify only the strongest and most abundant interactions; these approaches lack the temporal control necessary for profiling the dynamic interactome of bromodomains, whose *K*_d_ towards acetylated proteins vary from 10–100 µM.^9^

**Fig. 1:**
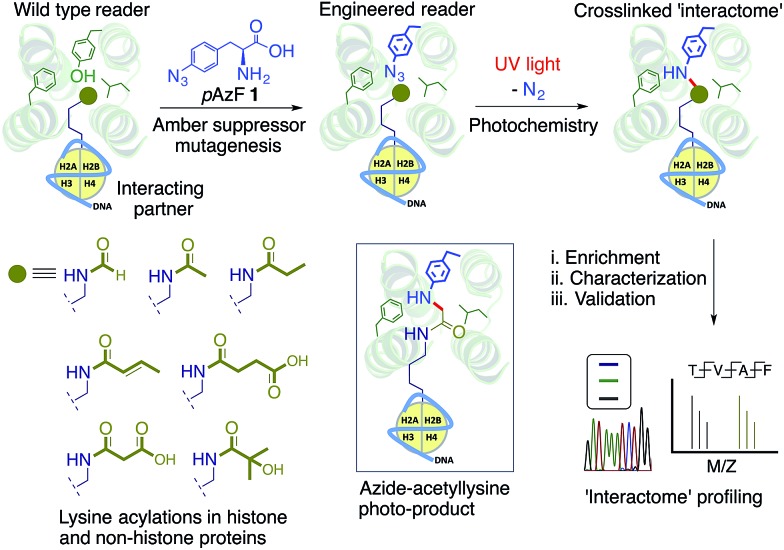
Schematic representation of the IBPP approach. The aromatic cage of a reader module is engineered to incorporate *p*-azido phenylalanine (*p*AzF) **1** without altering its affinity towards post-synthetically modified interacting partners such as acylated histone and non-histone proteins. Interacting partners are cross-linked and enriched using an affinity tag present in the reader and subsequently characterized and validated by western blotting, genomic and/or proteomic analysis. Shown in the inset is the crosslinked product of a photoreaction between the *p*AzF modified reader (*e.g.* bromodomain) and the acetylated lysine in histone.

We envisioned a novel approach based on protein interface engineering for characterizing the reader-specific interacting partners with high temporal precision (Fig. 1). The central tenet of our approach, which we term ‘interaction-based protein profiling’ (IBPP), is to incorporate a photo-sensitive amino acid deep inside the aromatic cage of a reader module to rapidly form a covalent bond with dynamic interacting partners upon exposure to ultra violet (UV) light.^10^ IBPP represents a substantial departure from the earlier approaches, as it shifts focus from simple peptide-based probes to full-length engineered readers capable of capturing even the transiently interacting, low abundance partners in cellular milieu.

To validate the IBPP approach, we focused on BRD4, a bromodomain and extra terminal (BET) containing protein.^11^ An epigenetic reader involved in gene regulation in diverse cellular processes,^9^ BRD4 is implicated in multiple malignancies due to its oncogenic translocation with the *NUT* gene and misregulated reading of acetylated non-histone proteins.^12^ Here we describe the engineering of BRD4 to include 4-azido-l-phenylalanine (*p*AzF, **1**)^13^ in its aromatic cage, validate the biochemical integrity of modified proteins, and show the ability of engineered BRD4 to crosslink to interacting proteins in a complex cellular mixture. We further establish the generality of this approach by applying it to other distantly related bromodomains. Finally, by uncovering and validating novel interacting partners identified through IBPP, we identify potential functions of BRD4 beyond its canonical role in transcription.

## Results and discussion

### Engineering the BRD4 bromodomain

**Fig. 2:**
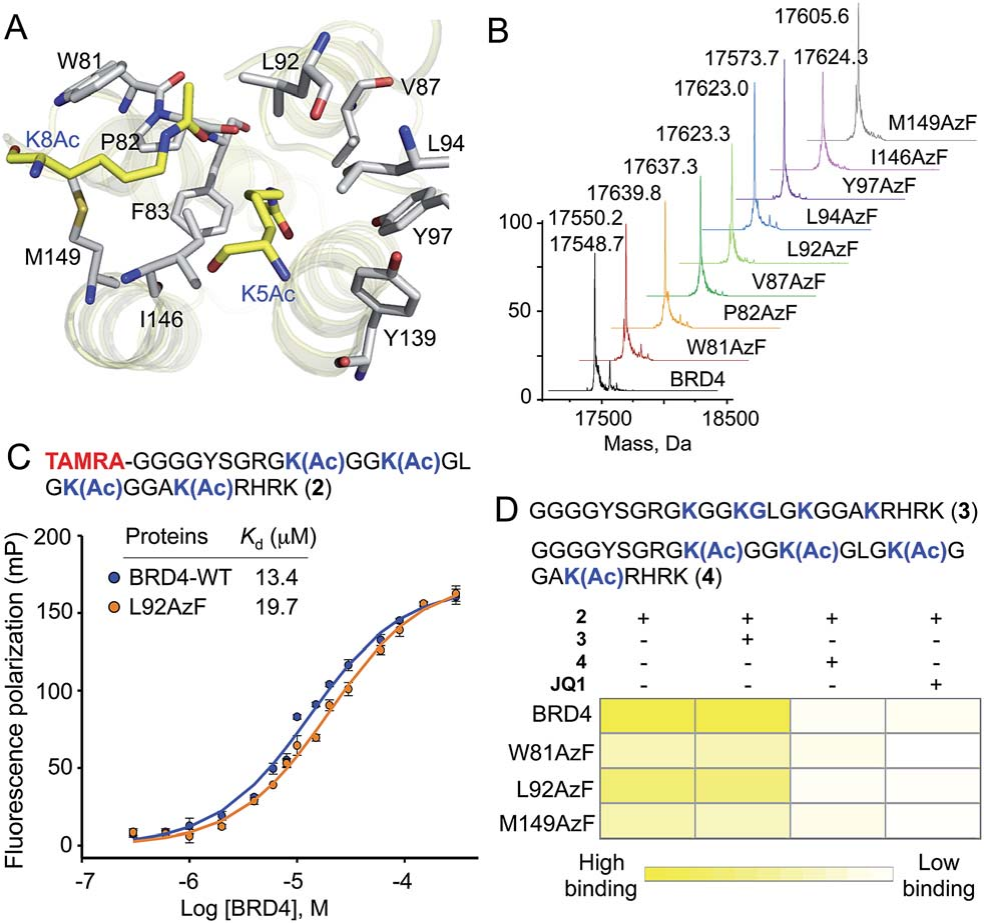
*p*AzF **1** containing BRD4 variants and their activity. (A) Crystal structure of BRD4 bromodomain showing multiple hydrophobic residues lining up to form an aromatic cage, essential for recognizing acetylated proteins. In the current study, each of these residues is targeted for replacement with *p*AzF. (B) LC-MS analysis of BRD4 mutants bearing *p*AzF. (C) Dissociation constants (*K*_d_) of BRD4 and its L92AzF mutant towards tetra-acetylated TAMRA-labeled H4 peptide **2** as measured by fluorescence polarization (FP). (D) Heat-map diagram showing binding (1/*K*_d_ in µM^–1^) of BRD4 and selected mutants towards **2** in the presence of unlabeled non-acetylated peptide **3**, and tetra-acetylated peptide **4**, and BRD4 inhibitor JQ1 using FP assay. *K*_d_ values are measured in triplicate.

Detailed structural studies on bromodomains have led to the identification of an aromatic cage formed by multiple hydrophobic residues that bind to acetylated lysine (Fig. 2A).^14^ Some of these residues can be altered without significant loss of binding affinity, particularly those that do not cause significant structural change. For instance, the Y139F mutation in BRD4 and the equivalent Y147F mutation in BPTF-bromodomain cause only a 2-fold loss in binding affinity towards acetylated histone peptide.^5,15^ Such observations led us to hypothesize that **1**, an analogue of phenylalanine, could be introduced into the BRD4 aromatic cage without significantly affecting the binding to its interacting partners. We focused on employing *p*AzF because of the minimal structural perturbation, high crosslinking efficiency and availability of suitable methods for introduction into proteins by both bacterial and mammalian expression.^13,16,17^

To site-specifically introduce **1** into BRD4, we employed amber suppressor codon (TAG) mutagenesis using an evolved orthogonal *M. jannaschii* TyrRS–tRNA_CUA_^Ty^ pair.^13^ Following a series of initial optimizations, we were able to express eight mutants (W81AzF, P82AzF, V87AzF, L92AzF, L94AzF, Y97AzF, I146AzF and M149AzF) in high yields (Fig. S1†). LC-MS analysis confirmed the integrity of the intact proteins bearing *p*AzF (Fig. 2B and Table S1†).

### Affinity of BRD4 mutants towards acetylated histone peptide

To examine the potential of the BRD4 mutants to bind acetylated substrates, we developed a fluorescence polarization (FP) assay with tetramethylrhodamine (TAMRA)-labeled tetra-acetylated histone peptide **2** (Fig. 2C and S2†).^15^ Wild-type BRD4 bound tracer peptide **2** with a *K*_d_ value of 13.4 µM, closely agreeing with reported values,^15,18^ while mutants displayed varied degrees of binding. Among the tested mutants, W81AzF, L92AzF and M149AzF showed comparable binding affinities with *K*_d_ values of 36.3, 19.7 and 32.8 µM, respectively (Fig. 2C and S2†). Significant loss of affinity was observed for the remaining mutants; P82AzF and Y97AzF were the least efficient, likely due to substantial structural changes for the former and loss of critical H-bonding for the latter. Consistently, mutations at Y97 disrupted the binding towards the peptide **2** (Fig. S3†).^15^ Collectively, we identified three *p*AzF-containing BRD4 mutants that are capable of recognizing the acetylated substrate with wild-type efficiency, thus demonstrating the feasibility of our engineering approach.

To verify that mutations did not change the integrity of the aromatic cage and the mode of binding, we tested the effect of bromodomain-specific small-molecule inhibitor JQ1 on binding.^19^ We observed that binding affinities of both wild type and mutant proteins towards peptide **2** were significantly reduced by the presence of 200 nM JQ1 (Fig. 2D and S4–7†). Given that JQ1 inhibited all the proteins to almost equal extent (9–11 fold reduction in binding), it is likely that the inhibitor binds wild type and mutants in a similar fashion. Further competition experiments with unlabeled non-acetylated (**3**) and tetra-acetylated (**4**) peptides revealed that only the latter could inhibit the binding of BRD4 and its selected mutants towards **2** (Fig. 2D and S4–7†).

To further establish that the mutations did not alter BRD4 substrate specificity, we synthesized a TAMRA-labeled tetra-acetylated peptide (**5**) with a random sequence (Fig. S8†). This control peptide has a similar length and lysine pattern as the histone peptide **2**. However, in FP-based binding assay, the random peptide did not show any affinity towards either wild-type BRD4 or selected mutants (Fig. S8†). This result further confirmed that the engineered reader with *p*AzF incorporated deep inside the aromatic cage displays native-like substrate specificity. Overall, the above results demonstrate that engineering the aromatic cage of BRD4 with *p*AzF does not change the biochemical integrity of the protein in terms of substrate affinity or specificity.

### Photo-crosslinking of engineered BRD4 with interacting partners

**Fig. 3:**
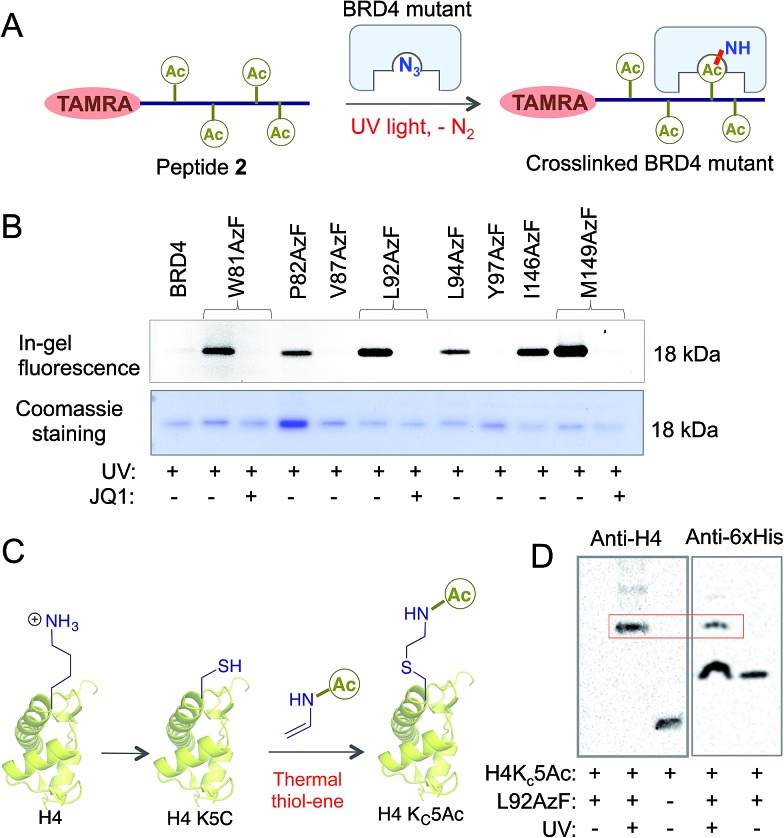
Crosslinking with histones. (A) Schematic showing binding of an engineered reader to the TAMRA-TetAc peptide **2** followed by crosslinking. Fluorescently labeled crosslinked readers are visualized by in-gel fluorescence as shown in (B). (B) Upon incubation with **2**, samples were exposed to 365 nm UV light followed by in-gel fluorescence using 532 nm light (*λ*_max_ for TAMRA). Samples that underwent successful photo-crosslinking upon exposure to 365 nm UV light are indicated by bands visible under 532 nm light. Crosslinking is completely abolished in the presence of 1 µM of JQ1. Coomassie staining of the same gel showed the presence of proteins in all the samples. The same amount of protein was used to perform each crosslinking experiment. (C) Scheme showing the synthesis of H4 Kc5Ac using a thermal thiol-ene reaction. (D) Visualization of crosslinking of H4Kc5Ac to L92AzF (shown in red box) using anti-H4 and anti-6xHis antibodies only when samples were exposed to UV light (lanes 2 and 4). H4 was not detected due to the lack of crosslinking in lane 1. Lane 3 shows only H4Kc5Ac as control.

Next we evaluated the potential of the engineered reader to crosslink to its interacting partners in the presence of UV light (Fig. 3A). TAMRA-labeled peptide **2** was incubated separately with wild type BRD4 and its mutants, followed by UV exposure. Subsequent enrichment with Ni-NTA beads *via* a His-tag on the proteins, extensive washing, and in-gel fluorescence revealed crosslinked bands of mutants and labeled peptides only upon exposure to UV light (Fig. 3B and S9†). The crosslinking ability of the mutants must have been acquired through active site engineering because fluorescent labeling of wild type BRD4 was undetectable in spite of its strong affinity towards the peptide. The crosslinking efficiency of mutant proteins corroborated well with their binding affinities towards the peptide (Fig. 2C, 3B and S2†). Observation that even a weak binder P82AzF could undergo crosslinking with the interacting partner highlights the potential of our approach to identify weak transient interactions that would be otherwise difficult for peptide-based pull-down methods. Importantly, the presence of 1 μM JQ1 completely abolished the crosslinking (Fig. 3B), demonstrating the specificity of binding and crosslinking through the engineered aromatic cage.

To demonstrate that the engineered reader is capable of interacting with and crosslinking to full-length binding partners, we generated thia-acetylayed histone H4 employing a thermal thiol-ene reaction to produce an acetylated Lys isostere (Fig. 3C).^20^ We generated Kc5Ac H4 protein by this method and confirmed its identity by LC-MS (Fig. S10†). The acetylated H4 underwent smooth crosslinking with L92AzF upon UV-irradiation and was successfully pulled-down using Ni-NTA beads. The crosslinked protein bands were visualized using anti-H4 and anti-6xHis antibodies (Fig. 3D). No crosslinked H4 was observed when not exposed to UV light.

### Generality of the bromodomain engineering approach

**Fig. 4:**
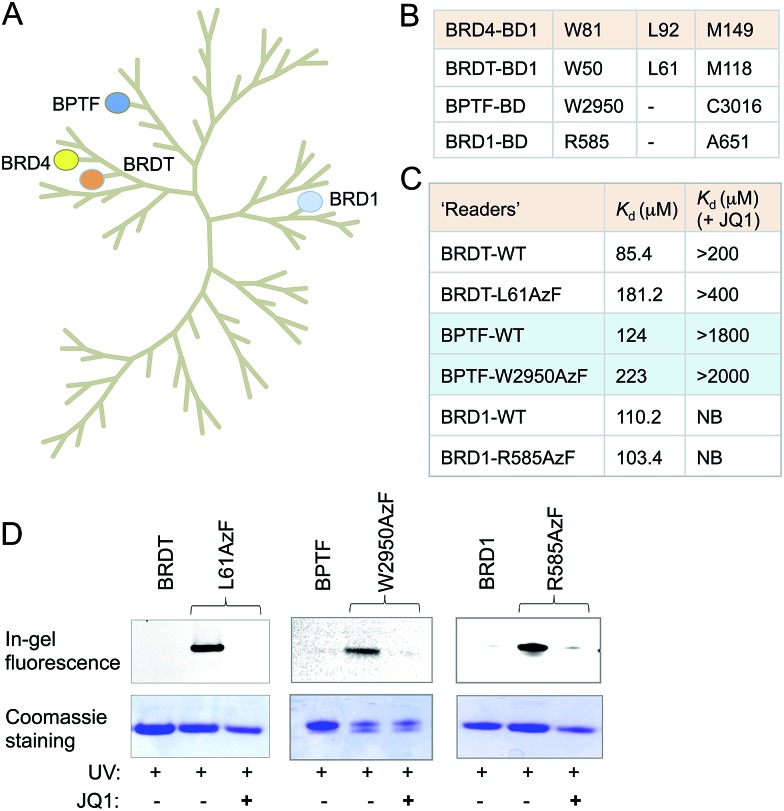
Generality of the IBPP approach. (A) Phylogenetic tree of the human bromodomain family. Proteins, represented with filled circles, have been subjected to IBPP in the current study. (B) W81, L92 and M149 residues of the first bromodomain (BD1) of BRD4 are conserved across bromodomains. (C) In the FP assay, BRDT-L61AzF, BPTF-W2950AzF and BRD1-R585AzF recapitulate the results of BRD4-L92AzF and BRD4-W81AzF mutants. Binding was strongly inhibited by JQ1. (D) In-gel fluorescence showing crosslinking of BRDT, BPTF, BRD1 and their mutants with TAMRA-TetAc peptide **2**. Crosslinking is completely abolished in the presence of JQ1. Coomassie staining of the same gel showed the presence of proteins in all the samples. *K*_d_ values are measured in triplicate (NB = no binding).

Sequence and structure alignment of multiple bromodomain-containing proteins revealed that their aromatic cages are conserved across protein families (Fig. 4A and S11†). To demonstrate the generality of our approach, we selected three distinct bromodomain-containing proteins BRD1, bromodomain testis-specific (BRDT) and BPTF.^5,21,22^ BRDT belongs to BET family of bromodomains like BRD4, while BRD1 and BPTF are non-BET members. BRDT and BPTF are essential for chromatin remodeling during spermatogenesis for male fertility and *Hox* gene expression, respectively.^5,21^ Although the biological functions of these bromodomain-containing proteins are beginning to emerge, characterization of their interacting partners remains mostly unexplored. Guided by our observations in the BRD4 system, we generated mutants of these three proteins: L61AzF BRDT (equivalent to L92AzF in BRD4), R585AzF BRD1 (equivalent to W81AzF in BRD4) and W2950AzF BPTF (equivalent to W81AzF in BRD4) (Fig. 4B). Protein identity and purity were confirmed in each case by LC-MS (Fig. S1, S12–14 and Table S1†). In a similar fluorescence polarization assay, we noticed that the *p*AzF mutants showed similar affinity as their wild-type counterparts towards tetra-acetylated histone peptide **2** (Fig. 4C and S12–14†). Importantly, the binding was abrogated in the presence of JQ1. This result supports our hypothesis that the conserved site inside aromatic cage can be engineered to extend the IBPP approach to multiple bromodomain-containing proteins.

We next performed photo-crosslinking experiments with wild type proteins (BRDT, BRD1 and BPTF) and their mutants (L61AzF, R585AzF and W2950AzF) using peptide **2** as described above (Fig. 4D). In the presence of UV light, the mutant proteins underwent smooth crosslinking with the bound acetylated peptide but not in presence of JQ1. In contrast, wild type proteins failed to undergo crosslinking with the peptide. Together, these data strongly argue for the generality of the IBPP approach in profiling interacting partners of a range of bromodomain-containing proteins.

### Application of IBPP to characterize the BRD4 interactome

**Fig. 5:**
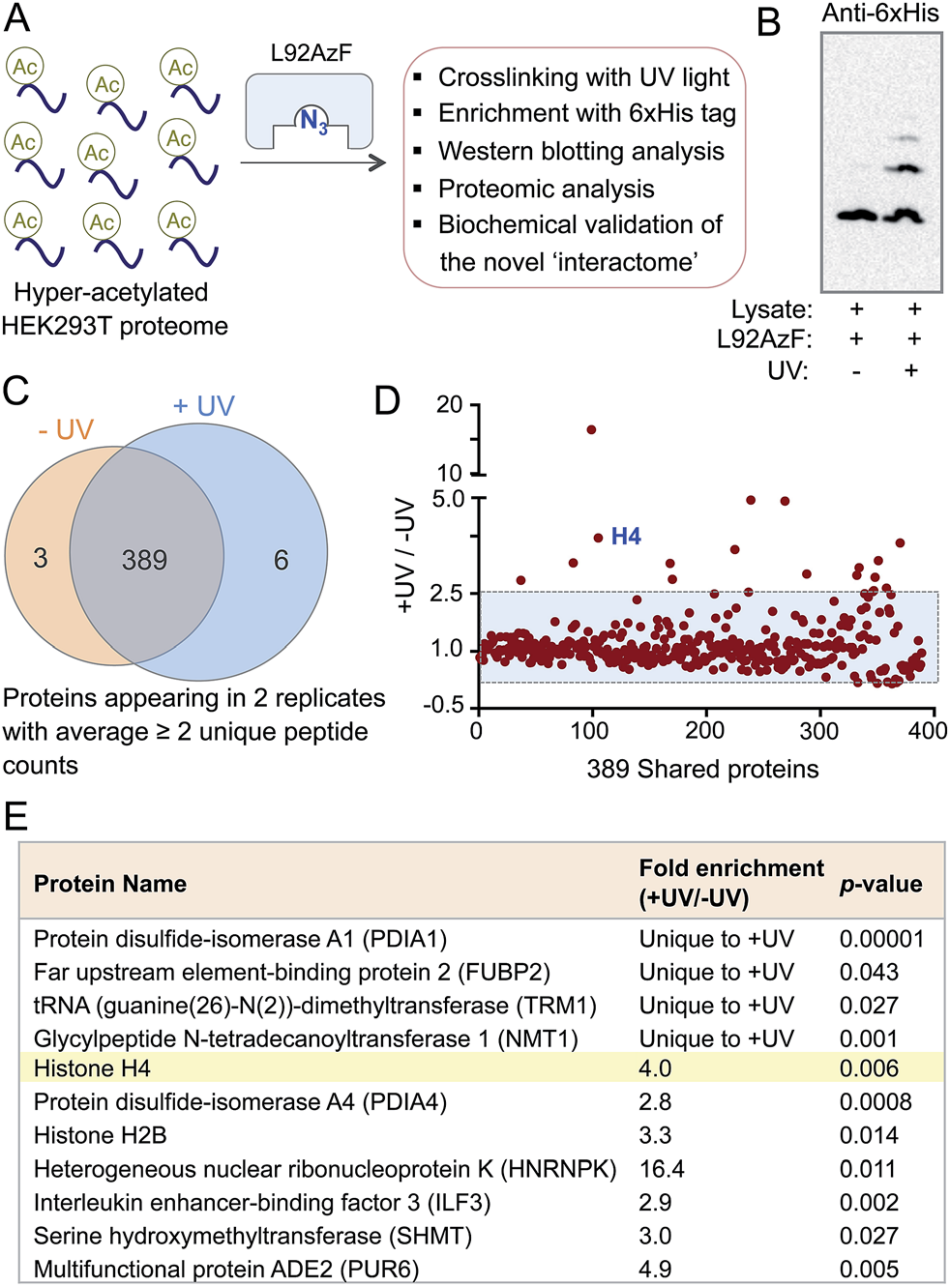
Crosslinking with cellular proteome. (A) Schematic representation of the pull-down of cross-linked interacting partners of BRD4 from cellular extracts. (B) Western blotting with anti-6xHis antibody demonstrated the presence of multiple crosslinked interacting proteins of L92AzF (lane 2). (C) Venn diagram showing stringently filtered datasets appearing in –UV and +UV treated samples as well as in both. (D) The enrichment ratios of the 389 shared proteins as judged by the normalized total counts were plotted against the unique target ID. The shaded area represents non-specific proteins. (E) High-confidence BRD4 interactome with *p*-values <0.05 as identified through IBPP.

The success in engineering BRD4 to bind and crosslink to acetylated histone H4 prompted us to further validate the IBPP approach in identifying proteome-wide interacting partners of the reader protein. To examine whether the engineered BRD4 is able to crosslink and enrich interacting proteins from a complex biological mixture, we generated hyper-acetylated human proteome in HEK293T cells using a deacetylase inhibitor (Fig. 5A).^23^ The lysate was incubated with L92AzF followed by crosslinking using UV light. Crosslinked proteins were pulled down with Ni-NTA beads and washed extensively. The eluted proteins were subjected to Western blotting using anti-His antibody. The presence of multiple higher molecular weight bands (Fig. 5B) suggested the crosslinking of the engineered reader to various interacting partners present in the cell lysate. No enrichment of these proteins was observed when the samples were not exposed to UV-irradiation. Furthermore, a competition experiment with increasing concentration of wild type BRD4 led to complete elimination of crosslinked protein bands (Fig. S15†), suggesting little or no off-target activity of the engineered BRD4.

The ability of IBPP to crosslink and enrich a series of proteins, as evident from Western blotting, led us to undertake mass spectrometry-based proteomic analysis to elucidate the constituents of this putative BRD4 interactome. The pulled-down proteins were resolved in SDS-PAGE and subjected to in-gel trypsin digestion.^24,25^ The tryptic peptides were subsequently characterized by liquid chromatography coupled with tandem mass spectrometry (LC-MS/MS). Samples that were not exposed to UV light served as negative controls in our experiment. The identified proteins from UV- and non UV-treated (*N* = 2 biological replicates for both cases) samples were compiled, and the exclusive unique peptides detected for each protein under each condition were averaged. Proteins that were present in both biological replicates and demonstrated a minimum of two unique peptides were selected for further analysis. Applying these criteria, we identified 398 proteins and placed them into three categories: 6 proteins were exclusive to UV-treated sample, 3 were exclusive to non UV-treated sample, and 389 were shared by both the samples (Fig. 5C). Further employing a ≥2.5-fold enrichment filter over the negative control led to the identification of fifteen proteins from the set of 389 to be significantly enriched in +UV-treated condition (Fig. 5D). Finally, a *t*-test between control and UV-treated samples generated a set of eleven proteins with a *p*-value of ≤0.05 as high-confidence BRD4 interaction partners (Fig. 5E). Such criterion excluded all but one protein in non UV-treated samples. Furthermore, histone H4 was specifically enriched (four-fold enrichment ratio with a *p*-value of 0.006) from the UV-treated sample (Fig. 5E), validating the IBPP approach to identify authentic interacting partners of epigenetic readers from complex cellular milieu.

Analysis of the proteins in this newly described BRD4 interactome revealed that they are implicated in diverse biological processes such as chromatin remodeling, posttranscriptional processing, immune response, protein folding and folate metabolism. These results suggest the functions of BRD4 expand far beyond transcription (Fig. 5E and Table S2†). The presence of proteins that are involved in chromatin-templated processes is consistent with the nuclear localization of BRD4. Representative examples are histone H2B (component of histone octamer), ILF3 (transcription factor), FUBP2 (DNA-binding protein) and HNRNPK (RNA binding protein and a transcription factor). The putative BRD4 partner also includes an RNA methyltransferase and protein disulfide isomerase. Interestingly, the IBPP-derived interactome covers some, but not all, of the known interacting partners of BRD4 such as NF-κB subunit RelA.^26^ It therefore remains to investigate whether the difference is simply due to the low abundance or absence of targets in HEK293T or in experimental conditions as demonstrated earlier that different deacetylase inhibitors establish non-overlapping ‘acetylome’ pattern in human cells.^4^ Collectively, employing IBPP, we have uncovered a range of interacting proteins of BRD4 that represent a much more diverse set of proteins compared to the limited numbers of interacting partners identified previously through candidate-based approaches.

### Validation of selected interacting partners with wild type BRD4

**Fig. 6:**
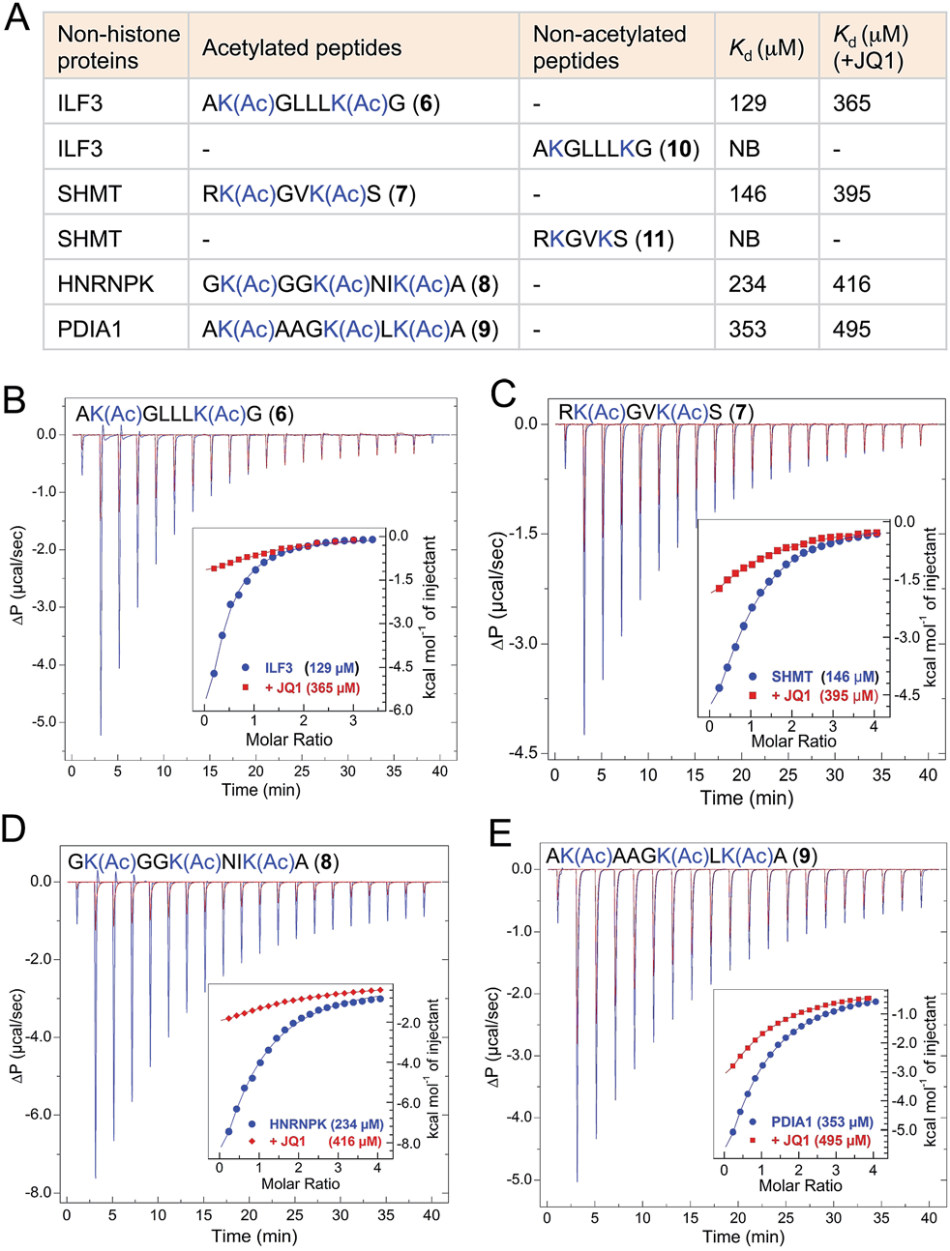
Biochemical validation of non-histone interactome of BRD4. (A) Table showing selected non-histone interacting partners of BRD4 identified through IBPP. Acetylated peptides corresponding to these proteins were subjected to isothermal titration calorimetry-based binding assay (B–E) to determine the dissociation constants of these peptides from wild type BRD4. JQ1 significantly inhibited these binding events as evident from increase in *K*_d_ values. *K*_d_ values are measured in triplicate (NB = no binding).

The majority of the proteins identified using IBPP are known to be acetylated in human cells as evident from recent proteomic studies.^4,27^ However the biological role of lysine acetylation in these proteins remain largely unexplored. To examine whether the functions of these acetylations could be mediated, at least in part, through BRD4, we selected four representative interacting partners identified through IBPP (∼50% of the high-confidence non-histone targets identified in the current study): ILF3, SHMT, HNRNPK and PDIA1 (Fig. 6). We synthesized short peptides **6–9** carrying multiple acetylated lysine residues many of which are known to be acetylated in these proteins in mammalian cells (Fig. 6A).^4,27^ In order to validate the interactions and obtain accurate binding constants of wild type BRD4 towards these peptides in solution, we determined the dissociation constants by isothermal titration calorimetry. The wild type BRD4 indeed recognized the acetylated peptides derived from these non-histone interacting partners as determined by *K*_d_ values that ranged from 120 to 340 µM (Fig. 6, Tables S3 and S4†). We further observed that, in a similar ITC assay, JQ1 significantly suppressed the binding of BRD4 towards the acetylated peptides, establishing the ability of IBPP to identify novel ‘interactomes’ of epigenetic readers. We also synthesized non-acetylated peptides **10–11** corresponding to ILF3 and SHMT as representative examples and observed no binding events with the wild type BRD4 (Fig. 6A, S16 and Table S3†), further confirming the interactome recognition exclusively through acetylated lysine residues.

Even though the affinity of the acetylated non-histone peptides was weaker compared to the histone peptide (120 to 340 µM *K*_d_ for the acetylated non-histone peptides compared to 20 µM for the acetylated H4 peptide) (Fig. 6, S17 and Tables S3, S4†), it’s certainly within the range in which diverse bromodomains bind their dedicated acetylated histones.^8^ In general, *in vitro* affinities of acetylated lysine for bromodomains are low, suggesting that additional interaction domains may be required for higher affinity target-specific binding *in vivo*. For example, the interaction of the acetylated transcription factor Twist to the second bromodomain of BRD4 is in the order of 800 µM.^28^ However, such low affinity is critical to recruit BRD4 which then interacts with the acetylated H4 through its first bromodomain to direct expression of Twist-dependent genes in certain breast cancer.^28^ Similarly, low *in vitro* interaction between acetylated RelA and only the first bromodomain of BRD4 plays a critical role in the expression of NF-κB dependent genes in human cells.^26,29^ Collectively, our results establish that IBPP-mediated newly uncovered non-histone proteins specifically interact with BRD4 through acetylated lysine residues and such interactions very likely will dictate the biological functions of BRD4 *in vivo*.

## Conclusions

Reader-mediated transcriptional programming is manifested through the combinatorial readout of DNA, histone, and non-histone modifications that cause transcriptional machinery to target specific genomic loci.^2^ To investigate such a complex network and identify interacting components, we have outlined an approach called IBPP involving protein engineering, UV-light mediated crosslinking and subsequent characterization of these components. As a proof-of-concept, we have successfully engineered multiple bromodomains using amber codon suppression to introduce an azide functionality that can be activated by light, an agent known to manipulate biology with high spatiotemporal precision.^30^ The potential of these engineered readers to interact with and crosslink to novel binding partners in complex cellular milieu was subsequently examined. Employing IBPP, we expanded the potential functions of BRD4 in diverse biochemical processes through the recognition of acetylated non-histone interacting partners such as transcription factors, chromatin regulators and proteins involved in RNA processing. To our knowledge, this current work represents the first example of reader-specific profiling of ‘acetylome’ (a collection of acetylated proteins) by setting up an azide-acetyllysine photoreaction deep inside the aromatic cage of bromodomains. We further biochemically validated a set of non-histone interacting partners to provide evidence that such non-canonical interactions may potentially regulate BRD4-mediated functions in chromatin remodeling, protein folding as well as in cellular metabolism. We anticipate that future experiments with full-length engineered readers expressed in mammalian cells and in-cell crosslinking will allow precise mapping of physiologically relevant interacting partners. Furthermore, a large number of human proteins carry reader domains (>60 bromodomains^31^ and ∼200 PHD fingers^32^) to recognize chemical modifications in histone and non-histone proteins. The molecular mechanisms by which these modules participate in diverse physiological processes (*e.g.* self-renewal,^33^ cell-cycle progression^34^ and oocyte–embryo transition^35^) are largely unknown. We envision extending the IBPP approach delineated here to shed light on the role of these readers in human biology and disease.
