# Supplementary material for: Site-specific azide-acetyllysine photochemistry on epigenetic readers for interactome profiling
Source: Chem Sci. 2017 Mar 14;8(6):4250–6. doi: 10.1039/c7sc00284j (PMC5468995; doi:10.1039/c7sc00284j)
Supplement: Supplementary file 1 [file SC-008-C7SC00284J-s001.pdf]

Electronic Supplementary Material (ESI) for Chemical Science.
This journal is © The Royal Society of Chemistry 2017

Site-Specific Azide-Acetyllysine Photochemistry on Epigenetic Readers for Interactome Profiling

Babu Sudhamalla, Debasis Dey, Megan Breski, Tiffany Nguyen, Kabirul Islam*
Department of Chemistry, University of Pittsburgh, Pittsburgh PA 15260

Contents

1. General materials, methods and equipment S2
2. Large-scale synthesis and characterization of *p*-azido-L-phenylalanine S3
3. Synthesis and purification of peptides S4
4. Expression and purification of BRD4-BD1 and its variants in *E. coli* S4
5. Fluorescence polarization (FP) binding assay with peptides S6
6. Photo-crosslinking experiment with peptides and in-gel fluorescence S7
7. Expression and purification of wild-type histone H4 and its variants S7
8. Chemical acetylation of cysteinylated histone H4 S8
9. Photo-crosslinking with full length acetylated histone H4 S8
10. Mammalian cell culture and cell lysis S9
11. Photo-crosslinking with HEK293T cell lysate S9
12. Western blotting S10
13. LC-MS analysis S10
14. LC-MS/MS analysis S11
15. Isothermal titration calorimetry S12
16. Supplementary figures and tables S13
17. References S29

1. General materials, methods and equipment

Chemicals: All chemicals were purchased from established vendors (e.g. Sigma-Aldrich, Acros Organics) and used without purification unless otherwise noted. Optima grade acetonitrile was obtained from Fisher Scientific and degassed under vacuum prior to HPLC purification. All reactions to prepare *p*AzF were carried out in round bottom flasks and stirred with Teflon®-coated magnetic stir bars under inert atmosphere when needed. Analytical thin layer chromatography (TLC) was performed using EMD 250 micron flexible aluminum backed, UV F_{254} pre-coated silica gel plates and visualized under UV light (254 nm) or by staining with phosphomolybdic acid, ninhydrin or anisaldehyde. Reaction solvents were removed by a Büchi rotary evaporator equipped with a dry ice-acetone condenser. Analytic and preparative HPLC was carried out on an Agilent 1220 Infinity HPLC with diode array detector. Concentration and lyophilization of aqueous samples were performed using Savant Sc210A SpeedVac Concentrator (Thermo), followed by Labconco Freeze-Dryer system.

Proton nuclear magnetic resonance spectra (1H NMR) were recorded on Bruker Ultrashield™ Plus 600/500/400/300 MHz instruments at 24 °C. Chemical shifts of ^{1}H and ^{13}C NMR spectra are reported as d in units of parts per million (ppm) relative to tetramethylsilane (δ 0.0) or residual solvent signals: chloroform-d (d 7.26, singlet), methanol-d_4 (δ 3.30, quintet), and deuterium oxide-d_2 (δ 4.80, singlet). Coupling constants are expressed in Hz. Mass spectra were collected at the UPITT MASSSPEC lab on a Q-Exactive™ Thermo Scientific LC-MS with electron spray ionization (ESI) probe.

Plasmids, mutagenic primers, cell lines and antibodies: All the plasmids are for bacterial expression and obtained as gifts from individual laboratories or purchased from Addgene. Mutagenic primers are obtained from Integrated DNA Technologies (**Table S5**). Competent bacterial cells used for protein expression and mutagenesis are given in **Table S6**. Human embryonic kidney 293T (HEK293T) cells, obtained from the American Type Culture Collection (ATCC) and used in the current study following manufacturer's protocol (details in the experimental section 10). All the antibodies used in the current study are purchased from established vendors and used following manufacturer's protocol (details in the experimental section 12).

2. Large-scale synthesis and characterization of *p*-azido-L-phenylalanine (*p*AzF, 1)[1]

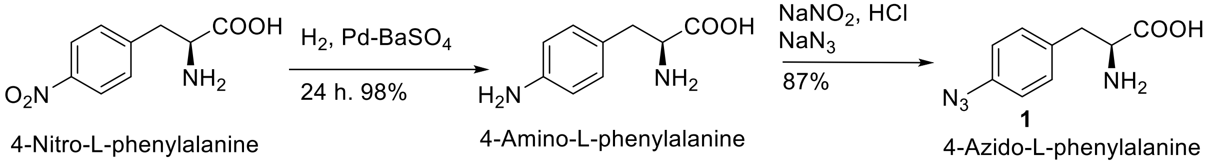

4-amino-L-Phenylalanine

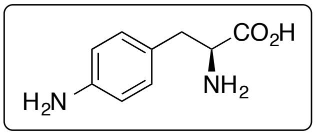

To a suspension of 4-Nitro-L-phenylalanine (5.0 g, 23.79 mmol) in 150 mL of water was added Pd-BaSO$_4$ (0.8 g, 10 mol%). The mixture was degassed under vacuum (5 min), fitted with a hydrogen balloon. The mixture was stirred at rt. for 24 hr. and filtered through a pad of Celite. The filtrate was concentrated under reduced pressure to obtain 4-Amino-L-phenylalanine (4.2 g, 98%) as yellowish solid.

^{1}H NMR (D_2O, 400MHz): δ = 7.07 (d, *J*=8.4 Hz, 2 H), 6.73 - 6.84 (d, *J*=8.4 Hz, 2 H), 3.87 (dd, *J*=7.8, 5.1 Hz, 1 H), 3.12 (dd, *J*=14.7, 5.1 Hz, 1 H), 2.96 (dd, *J*=14.7, 7.8 Hz, 1 H) ppm

^{13}C NMR (D_2O, 150MHz): δ = 174.1, 145.4, 130.3, 125.6, 116.8, 56.1, 35.5 ppm

HRMS m/z $[M+H]^+$: Calcd. for $C_9H_{13}N_2O_2$: 181.0971, found. 181.0960

4-azido-L-Phenylalanine (1)

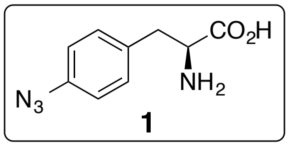

To an ice cold stirred solution of 4-Amino-L-phenylalanine (1.0 g, 5.55 mmol) in HCl (2.77 mL, 6M) was added an aq. solution of NaNO$_2$ solution (0.383 g, 5.55 mmol, dissolved in 0.5 mL water) added dropwise. After the addition, the reaction mixture was stirred for 10 min in an ice bath. Additional HCl (4.6 mL, 6M) was added, followed by addition of NaN$_3$ solution (0.342 g, 5.3 mmol, dissolved in 1 mL water, added 0.05 mL at a time). The reaction mixture was stirred for additional 15 min, after which the ice bath was removed. The reaction mixture was allowed to stir at rt. for an hour. EtOAc (20 mL) was added and the reaction mixture was filtered. The precipitate was washed with EtOAc (3x10 mL) and dried under vacuum to give **1** (4-Azido-L-phenylalanine, 1.0 g, 87%) as light yellowish solid.

^{1}H NMR (D_2O, 400MHz): δ = 7.31 (d, *J*=8.5 Hz, 2 H), 7.10 (d, *J*=8.5 Hz, 2 H), 4.22 (dd, *J*=7.6, 5.7 Hz, 1 H), 3.24 - 3.34 (m, 1 H), 3.10 - 3.23 (m, 1 H) ppm

^{13}C NMR (D_2O, 150MHz): δ = 171.2, 139.5, 130.9, 130.4, 119.5, 53.9, 34.9 ppm

HRMS m/z $[M+H]^+$: Calcd. for $C_9H_{11}N_4O_2$: 207.0876, found: 207.0864

3. Synthesis and purification of peptides

All the peptides were synthesized by the University of Pittsburgh Peptide Synthesis Facility. Analytical-scale separation was performed using ZORBAX reversed-phase C18 (5μm, 4.6 × 250 mm) column with UV detection at 280 nm. The column was equilibrated with 0.1% aqueous trifluoroacetic acid solution prior to each injection. Analytical separation was performed with a linear gradient of acetonitrile to 10% in 15 min and then to 70% in 5 min in 0.1% aqueous trifluoroacetic acid with a flow rate of 1 mL/min. The crude peptides were purified using preparative reversed-phase HPLC (XBridge C18, 5 μm, 10 x 250 mm column) eluting with a flow rate of 4 mL/min and a gradient of acetonitrile starting from 5% to 60% in 13 min and then to 100% in 7 min in aqueous trifluoroacetic acid (0.01%). The purified peptides were first concentrated by SpeedVac concentrator followed by lyophilization. The dried peptides were resuspended in water containing 0.01% TFA and stored at -20 ^{o}C before use. Concentrations of the TAMRA-labeled acetylated peptides **2** and **5** were determined by UV absorption with ε_{555} = 65000 $L^{-1}cm^{-1}M^{-1}$. Concentrations of unlabeled non-acetylated (**3**) and tetra-acetylated (**4** and **12**) histone H4 peptides as well as acetylated non-histone peptides (**6**-**11**) were determined based on the observation that 1 mg/ml peptide generates an absorbance value (A_{205}) of 30 at 205 nm. The integrity of the purified peptide was confirmed by MALDI mass spectrometry.

4. Expression and purification of BRD4-BD1 and its variants in *E. coli*

The N-terminal 6xHis-tagged human BRD4-BD1 (first bromodomain of BRD4) expression construct pNIC28-Bsa4 (Addgene ID: 38942) and evolved *Methanococcus Jannaschii p*-Azido-L-phenylalanine RS (2 copies+tRNA) expression vector pEVOL-pAzF (Addgene ID: 31186)[2] were obtained from Addgene. The wild type BRD4-BD1 plasmid was transformed in to *E. Coli* BL21 codon plus (DE3) RIPL competent cells (Invitrogen) using pNIC28-Bsa4 kanamycin-resistant vector.[3] A single colony was picked up and grown overnight at 37 ^{o}C in 10 mL of Luria-Bertani (LB) broth in presence of 50 μg/mL kanamycin and 35 μg/mL chloramphenicol. The culture was diluted 100-fold and allowed to grow at 37 ^{o}C to an optical density

(OD_{600}) of 0.8, and protein expression was induced overnight at 18 ^{o}C with 0.5 mM IPTG in an Innova 44® Incubator shaker (New Brunswick Scientific). Proteins were purified as follows: harvested cells were resuspended in 15 mL lysis buffer (50 mM Tris-HCl pH 8.0, 150 mM NaCl, 5 mM β-mercaptoethanol, 10% glycerol, 25 mM imidazole, Lysozyme, DNase, and Roche protease inhibitor cocktail). The cells were lysed by pulsed sonication (Qsonica-Q700), and centrifuged at 13000 rpm for 40 min at 4 ^{o}C. The soluble extracts were subject to Ni-NTA agarose resin (Thermo) according to manufacturer's instructions. After passing 20 volumes of washing buffer (50 mM Tris-HCl pH 8.0, 150 mM NaCl, 5 mM β-mercaptoethanol, 10% glycerol, and 25 mM imidazole), proteins were eluted with a buffer containing 50 mM Tris-HCl pH 8.0, 150 mM NaCl, 5 mM β-mercaptoethanol, 10% glycerol, and 400 mM imidazole. Proteins were further purified by gel filtration chromatography (Superdex-75) using AKTA pure FPLC system (GE healthcare) with buffer containing 50 mM Tris-HCl pH 8.0, 150 mM NaCl, and 10% glycerol. Purified proteins were concentrated using Amicon Ultra-10k centrifugal filter device (Merck Millipore Ltd.). The protein concentration was determined using Bradford assay kit (BioRad Laboratories) with BSA as a standard. The concentrated proteins were stored at -80 ^{o}C before use.

Point mutation was introduced into the pNIC28-Bsa4 BRD4-BD1 construct to generate Y97A mutant using the QuikChange Lightning site-directed mutagenesis kit (Agilent Technologies) following manufacturer's protocol using ARKTIK Thermal cycler-5020 (Thermo Scientific). The resulting mutant plasmids were confirmed by DNA sequencing. All the mutants were expressed and purified following the procedure used for wild type BRD4-BD1 protein.

The wild-type BRD4-BD1 gene in the vector was mutagenized using the QuikChange Lightning site-directed mutagenesis kit (Agilent Technologies) to generate a series of clones with an amber suppressor codon (TAG) at positions W81, P82, F83, V87, L92, L94, Y97, C136, Y139, D144, D145, I146 and M149. The resulting mutant plasmids were confirmed by DNA sequencing. To express BRD4 variants carrying *p*-Azido-L-phenylalanine (*p*AzF) at specific positions, we co-transformed *E. Coli* BL21 star (DE3) cells (Invitrogen) with pEVOL-pAzF and BRD4-BD1 Amber variants (W81TAG, P82TAG, F83TAG, V87TAG, L92TAG, L94TAG, Y97TAG, C136TAG, Y139TAG, D144TAG, D145TAG, I146TAG, M149TAG). Cells were recovered in 200 µL of the SOC medium for 1 h at 37 ^{o}C before being plated on a LB agar plate containing Kanamycin (Kan) (50 µg/mL) and Chloramphenicol (Cm) (35 µg/mL). A single colony was selected and grown at 37 ^{o}C in 10 mL LB broth in presence of 50 µg/mL Kan and 35 µg/mL Cm overnight. The overnight culture was centrifuged for 10 min at 1000g. 8 mL of LB broth was subsequently removed and the cell pellet with remaining 2 mL LB broth was used to inoculate 1 L of the GMML medium supplemented with 35 µg/mL Cm and 50 µg/mL Kan. Cells were allowed to grow at 37 ^{o}C in an incubator

shaker (225 rpm) until OD_{600} reached to ~0.8. *p*AzF was aseptically added to the culture to a final concentration of 1 mM and the culture was cooled to 17 ^{o}C for 30 min while shaking at 225 rpm. The culture was then induced with 0.05 % arabinose and continued to shake at 17 ^{o}C for an additional 30 min. Finally, the protein expression was induced by the addition of 0.25 mM IPTG and allowed to shake at 225 rpm for 20 h at 17 ^{o}C. All the mutant proteins were purified as described above for the wild type BRD4-BD1 protein.

5. Fluorescence polarization (FP) binding assay with peptides

Fluorescence polarization assay was performed to measure binding of the wild type BRD4-BD1 and its mutants to (TAMRA)-labeled tetra-acetylated H4 peptide **2**. The experiments were performed in 384-well small volume black/clear microtiter plates (Falcon) with 200 nM TAMRA-labeled peptide and varying concentrations of proteins (0.3 to 800 μM) in a buffer containing 10 mM Tris-HCl pH 7.5, 150 mM NaCl, 0.05% Tween 20, and 0.5 mM Tris (2-carboxyethyl) phosphine (TCEP). After 30 min of incubation at room temperature, FP was measured in a Tecan M1000 plate reader using its FP module and at excitation/emission wavelengths of 530 and 570 nm, respectively. For determinations of the dissociation constants (K_d), the background corrected polarization values were plotted against the concentrations of proteins. The data were fitted to a single-site binding equation $Y = B_{max} X / (K_d + X)$, where Y is the specific binding, B_{max} is the maximal binding and X is the concentration of the ligand, using the SigmaPlot software.[3]

For competition assays, 250 μM of unlabeled non-acetylated (**3**) and tetra-acetylated (**4**) H4 peptide and 200 nM JQ1 were separately preincubated with varying concentrations of BRD4-BD1 and its variants W81AzF, L92-AzF and M149AzF (0.3 to 800 μM) in a buffer containing 10 mM Tris-HCl pH 7.5, 150 mM NaCl, 0.05% Tween 20, and 0.5 mM Tris (2-carboxyethyl) phosphine (TCEP). After 30 min of incubation at room temperature, 200 nM TAMRA-labeled tetra-acetylated H4 peptide **2** was added followed by another 30 min incubation at room temperature. Fluorescence polarization was subsequently measured in a Tecan M1000 plate reader as described above. Following the same assay conditions including protein and peptide concentrations as described for the histone peptide **2**, fluorescence polarization assay was performed to measure binding of BRD4-BD1 and its L92AzF mutant to (TAMRA)-labeled tetra-acetylated random peptide **5**.

6. Photo-crosslinking experiment with peptides and in-gel fluorescence

For photo-crosslinking experiments, 1 μM TAMRA-labeled tetra-acetylated H4 peptide **2** was preincubated with 25 μM of wild type BRD4-BD1 or its variants in a buffer containing 10 mM Tris-HCl pH 7.5, 150 mM NaCl, 0.05% Tween 20, and 0.5 mM TCEP. After 30 min of incubation at room temperature, samples were subjected to UV irradiation at 365 nm for 15 min at 4 ^{o}C using Transilluminator 2040 EV (Stratagene). Proteins were then bound to Ni-NTA agarose resin pre-equilibrated with binding buffer (50 mM Tris-HCl, pH 8.0, 150 mM NaCl, 5 mM β-mercaptoethanol, and 25 mM imidazole) by gentle shaking at room temperature for 40 min. To remove the unbound peptide, the samples were washed 10 times with washing buffer (50 mM Tris-HCl pH 8.0, 200 mM NaCl, 1% Triton X-100). The crosslinked species were eluted with a buffer containing 50 mM Tris-HCl pH 8.0, 150 mM NaCl, 5 mM β-mercaptoethanol, and 400 mM imidazole. The eluted protein samples were separated on a 4-12 % Criterion XT precast gel (Bio-Rad Laboratories) and imaged on a ChemiDoc MP Imaging system using TAMRA fluorophore excitation wavelength (Emission filter 605/50, Light: green Epi illumination). The gel was subsequently stained with coomassie brilliant blue R-250 staining solution to confirm the presence of proteins in all the samples.

For Inhibition studies, selected BRD4-BD1 mutants W81AzF, L92AzF and M149AzF were preincubated with 1 μM of JQ1 for 30 min. Then added 1 μM of the TAMRA-labeled H4-teteraacetylated peptide **2** and incubated for an additional 30 min. Subsequent crosslinking, pull-down of crosslinked proteins and in-gel fluorescence were performed as described above.

7. Expression and purification of wild-type histone H4 and its variants

Gene sequence encoding wild type Xenopus laevis histone H4 was a kind gift from Dr. Minkui Luo at the Memorial Sloan-Kettering Cancer Center. The plasmid containing wild type Histone H4 was transformed into BL21 codon plus (DE3) RIPL competent cells. A single colony was picked up and grown overnight at 37 ^{o}C in 10 mL of LB broth with 100 μg/mL ampicillin and 35 μg/mL chloramphenicol. The inoculation culture was diluted 1:100 fold in fresh LB medium and cells were grown at 37 ^{o}C until OD_{600} reached to ~0.7. Protein expression was induced by the addition of 0.3 mM IPTG followed by growing for an additional 3 h at 37 ^{o}C. Cells were harvested by centrifugation at 5000 rpm for 30 min, and then resuspension of the pellet in 5 mL of lysis buffer (10 mM Tris-HCl pH 7.5, 2 M guanidinium hydrochloride (GdnHCl), 5 mM β-mercaptoethanol, 10% glycerol, DNase, Lysozyme and Roche protease inhibitor

cocktail). The cells were lysed by pulsed sonication and centrifuged at 20,000g for 40 min at 4 °C. Insoluble histone was recovered from inclusion bodies by dissolving in 6 M GdnHCl and 10 mM Tris-HCl pH 7.5, and incubated for 10 min at room temperature followed by centrifugation at 20,000g for 40 min at 4 °C. The soluble histone supernatant was purified by size exclusion chromatography on a Superdex-200 using AKTA pure FPLC system. Fractions were concentrated using Amicon Ultra-4 centrifugal 3K filter and further purified with preparative reversed-phase HPLC (XBridge C18, 5 µm, 10 x 250 mm column) eluting with a flow rate of 4 mL/min starting from 10% acetonitrile to 70 % in 15 min and then to 100 % over 5 min in aqueous trifluoroacetic acid (0.01%). The purified protein was concentrated by SpeedVac followed by lyophilization. The protein was stored at -20 °C before use. Histone H4 mutant K5C was generated by the QuikChange Lightning site-directed mutagenesis kit (Agilent Technologies) following manufacturer's protocol. The resulting mutant plasmid was confirmed by DNA sequencing. The K5C mutant was expressed and purified as described above for the wild type Histone H4.

8. Chemical acetylation of cysteinylated histone H4

Purified H4 K5C (0.5 mg) was dissolved in 375 µL of 6 M GdnHCl and 200 mM sodium acetate at pH 6.0.[4] To this solution was added 75 mM glutathione, 250 mM *N*-vinylacetamide, 500 mM dimethylsulfide, and 250 mM of the azo radical initiator VA-044 (2,2̀ -azobis[2-(2-imidazolin-2-yl) propane]dihydrochloride). The reaction was initiated by incubating the above mixture in the dark at 37 °C for 1 h. The product was purified by RP-HPLC as described above in section 8 and characterized by ESI LC-MS.

9. Photo-crosslinking with full length acetylated histone H4

For photo-crosslinking experiments, 10-12 µM histone H4 Kc5Ac protein was preincubated with 100 µM of L92AzF in a buffer containing 10 mM Tris-HCl pH 7.5, 150 mM NaCl, 0.05% Tween 20, and 0.5 mM TCEP. After 30 min of incubation at room temperature, samples were subjected to UV irradiation at 365 nm for 30 min at 4 °C. Negative controls were not subjected to UV irradiation. Samples were then bound to Ni-NTA agarose resin and incubated for 1 h at room temperature with gentle agitation. To remove uncrosslinked H4 Kc5Ac, samples were washed with washing buffer (50 mM Tris-HCl pH 8.0, 200 mM NaCl, 5% Triton X-100) 10 times; during each washing step the samples were incubated at 60 °C for 5 min. Finally cross-linked proteins were eluted with a buffer containing 50 mM Tris-HCl pH 8.0, 150 mM

NaCl, 5 mM β-mercaptoethanol, and 400 mM imidazole. The eluted proteins were separated on a 4-12 % Criterion XT precast SDS-PAGE gel (Bio-Rad Laboratories) and analyzed by Western blotting.

10. Mammalian cell culture and cell lysis

Human embryonic kidney (HEK) 293T cells were grown in Dulbecco modified Eagle medium (DMEM) (Gibco) supplemented with 10% fetal calf serum in a humidified atmosphere containing 5% CO_2 in a T150 flask. At 60-70% confluence stage, cells were treated with 5 μM of histone deacetylase inhibitor suberanilohydroxamic acid (SAHA, cat#10009929, Cayman chemical company) dissolved in DMSO to generate hyperacetylated histones.[5] Twelve hours post treatment, cells were harvested and lysed with 600 mL of cold RIPA buffer (Sigma) supplemented with 1X Roche protease inhibitor cocktail and 5 mM TCEP by sonicating for 15 min at amplitude of 60 with a repeating 20 sec pulse cycle. Cell lysates were centrifuged at 21 000*g* for 30 min at 4 °C to remove cell debris. The supernatant was then passed through the detergent removal spin column (Pierce, cat. no. 87778) and eluted with Tris buffer (50 mM Tris-HCl pH 8.0, 10% glycerol, 2 mM TCEP, 1X Roche protease inhibitor cocktail) following manufacturer's protocol. Protein concentration was determined by Bradford assay (Bio-Rad Laboratories). This stock solution was used for subsequent photo-crosslinking and Western blotting experiments.

11. Photo-crosslinking with HEK293T cell lysate

For photo-crosslinking studies, ~1 mg of HEK293T cell lysates were incubated with 50 μM of BRD4-L92AzF in a buffer containing 10 mM Tris-HCl pH 7.5, 150 mM NaCl, 0.05% Tween 20, and 0.5 mM TCEP. After 1 h of incubation at room temperature, the samples were subjected to UV irradiation at 365 nm for 30 min at 4 ^{o}C. Negative controls were not subjected to UV exposure. Samples were then bound to Ni-NTA agarose resin and incubated for 1 h at 4 ^{o}C with gentle rotation. To remove un-crosslinked proteins present in cell lysates, samples were washed with washing buffer (50 mM Tris-HCl pH 8.0, 400 mM KCl, 5% Triton X-100). During each washing step samples were incubated at 60 ^{o}C for 5 min. Finally the proteins were eluted with a buffer containing 50 mM Tris-HCl pH 8.0, 150 mM NaCl, 5 mM β-mercaptoethanol, and 400 mM imidazole. The eluted proteins were separated on a 4-12% Criterion XT precast SDS-PAGE gel (Bio-Rad Laboratories) and analyzed by Western blotting and tandem mass spectrometry.

12. Western blotting

Equal volumes of the pulled-down, photo-crosslinked samples (both for histone H4 and cell lysate) were separated on SDS-PAGE and transferred onto a 0.2 µm supported nitrocellulose membrane (Bio-Rad Laboratories) at a constant voltage of 40 for 2.5 h at 4 ^{o}C. Membranes were blocked with 20 mL of TBST buffer (50 mM Tris HCl pH 7.4, 150 mM NaCl, 0.01% Tween-20) with 5% nonfat dry milk for 1 hr. at room temperature with gentle shaking. The blocking buffer was then removed and membranes were washed with 20 mL of TBST buffer. For experiments with histone-H4 Kc5Ac, immunoblotting was performed with 1:300 diluted primary antibody (Histone-H4 mAb, cat# 61521, Active Motif) for 12 h at 4 ^{o}C. For cell lysate photo-crosslinking samples, immunoblotting was carried out with 1:200 diluted primary antibody (His-probe, cat# sc-8036, Santa Cruz Biotechnology, Inc.) for 12 h at 4 ^{o}C. The antibody solutions were removed and membranes were washed three times with TBST buffer. The blots were then incubated with HRP-conjugated secondary antibody Goat anti-Mouse IgG (cat#15014, Active motif) with 5% nonfat dry milk (1:10000 dilution) in TBST for 1 h at room temperature. After similar washing, protein bands were visualized by chemiluminescence using VISIGLO HRP Chemiluminescent substrates A and B (cat# N252-120ML and N253-120ML, aMReSCO) following manufacturer's protocol.

13. LC-MS analysis

Intact protein analysis using capillary LC ESI–TOF: Protein samples were loaded onto a PLRP-S column (Higgins Analytical, 5 µm, 1000A, 300 µm i.d.× 100 mm) of the LC system (Ultimate 3000, Dionex, Sunnyvale, CA) online coupled to an electrospray ionization (ESI) time-of-flight mass spectrometer (micrOTOF, BrukerDaltonics, Billerica, MA). Chromatographic separation was performed at a constant flow rate of 3.5uL/ min using a binary solvent system (solvent A: water with 2.5% acetonitrile and 0.1% formic acid; solvent B: acetonitrile with 0.1% formic acid) and a linear gradient program (0-5 min, 5% B; 5-10 min, 5-30% B; 10-35 min, 30-70% B; 35-44 min, 70% B; 44-45 min, 70-5% B; 45-60 min, 5% B). Mass spectra were acquired in positive ion mode over the mass range m/z 50 to 3000. ESI spectra were deconvoluted to obtain molecular ion masses with Data Analysis 3.3 (Bruker Daltonics, Billerica, MA) using the MaxEnt algorithm resulting in a mass accuracy of 0.01%.

14. LC-MS/MS analysis[6]

In gel trypsin digestion. In gel trypsin digestion was carried out as previously described.[22] Excised gel bands were washed with HPLC water and destained with 50% acetonitrile (ACN)/25mM ammonium bicarbonate until no visible staining. Gel pieces were dehydrated with 100% ACN, reduced with 10mM dithiothreitol (DTT) at 56°C for 1 hour, followed by alkylation with 55mM iodoacetamide (IAA) at room temperature for 45min in the dark. Gel pieces were then again dehydrated with 100% ACN to remove excess DTT and IAA, and rehydrated with 20ng/μl trypsin/25mM ammonium bicarbonate and digested overnight at 37°C. The resultant tryptic peptides were extracted with 70% ACN/5% formic acid, vacuum dried and re-constituted in18μl 0.1% formic acid.

Tandem mass spectrometry. Proteolytic peptides from in gel trypsin digestion were analyzed by a nanoflow reverse-phased liquid chromatography tandem mass spectrometry (LC-MS/MS). Tryptic peptides were loaded onto a C18 column (PicoChip™column packed with 10.5cm Reprosil C18 3μm120Å chromatography media with a 75μm ID column and a 15μm tip, New Objective, Inc., Woburn, MA) using a Dionex HPLC system (Dionex Ultimate 3000, ThermoFisher Scientific, San Jose, CA) operated with a double-split system (Personal communication with Dr. Steve Gygi from Department of Cell Biology, Harvard Medical School) to provide an in-column nano-flow rate (~300nl/min). Mobile phases used were 0.1% formic acid for A and 0.1% formic acid in acetonitrile for B. Peptides were eluted off the column using a 52 minute gradient (2-40% B in 42 min, 40-95% B in 1min, 95% B for 1 min, 2% B for 8 min) and injected into a linear ion trap MS (LTQ-XL,ThermoFisher Scientific) through electrospray.

The LTQ XL was operated in a date-dependent MS/MS mode in which each full MS spectrum [acquired at 30000 automatic gain control (AGC) target, 50ms maximum ion accumulation time, precursor ion selection range of m/z 300 to1800] was followed by MS/MS scans of the 5 most abundant molecular ions determined from full MS scan (acquired based on the setting of 1000 signal threshold, 10000 AGC target, 100ms maximum accumulation time, 2.0 Da isolation width, 30ms activation time and 35% normalized collision energy). Dynamic exclusion was enabled to minimize redundant selection of peptides previously selected for CID.

Peptide identification by database search. MS/MS spectra were searched using MASCOT search engine (Version 2.4.0, Matrix Science Ltd) against the UniProt human proteome database. The following modifications were used: static modification of cysteine (carboxyamidomethylation, +57.05 Da), variable modification of methionine (oxidation, +15.99Da). The mass tolerance was set at 1.4Da for the precursor ions and 0.8 Da for the fragment ions. Peptide identifications were filtered using PeptideProphet™ and

ProteinProphet® algorithms with a protein threshold cutoff of 99% and peptide threshold cutoff of 90% implemented in Scaffold™ (Proteome Software, Portland, Oregon, USA).

15. Isothermal titration calorimetry

Isothermal titration calorimetry (ITC) was carried out on an ITC_{200} instrument (MicroCal, Malvern). Experiments were conducted at 15°C, while stirring at 750 rpm. Buffers of protein and peptides were matched to 50 mM Tris-HCl, pH 8.0 and 200 mM NaCl. Each titration comprised 1 initial injection of 0.4 µL lasting 0.8s, followed by19 injections of 2 µL lasting 4s each at 2 min intervals. The initial injection was discarded during data analysis. The micro syringe (40 µL) was loaded with a solution of the peptide sample at a concentration of 4-5 mM and it was injected into the cell (200 µL), occupied by a protein at a concentration of 200-250 µM. For inhibition experiments 1-2 µM of JQ1 was incubated with BRD4 protein (200-250 µM) for 30 min at 15°C and then titrated with a solution of the peptide sample at a concentration of 4-5 mM. All the data was fitted to a single binding site model using the Microcal ITC_{200} Origin data analysis software to yield enthalpies of binding (ΔH) and binding constants (K_aS). Further thermodynamic parameters i.e. changes in entropy ΔS, changes in free energy ΔG and dissociation constants (K_dS) were calculated from these values.

16. Supplementary figures and tables

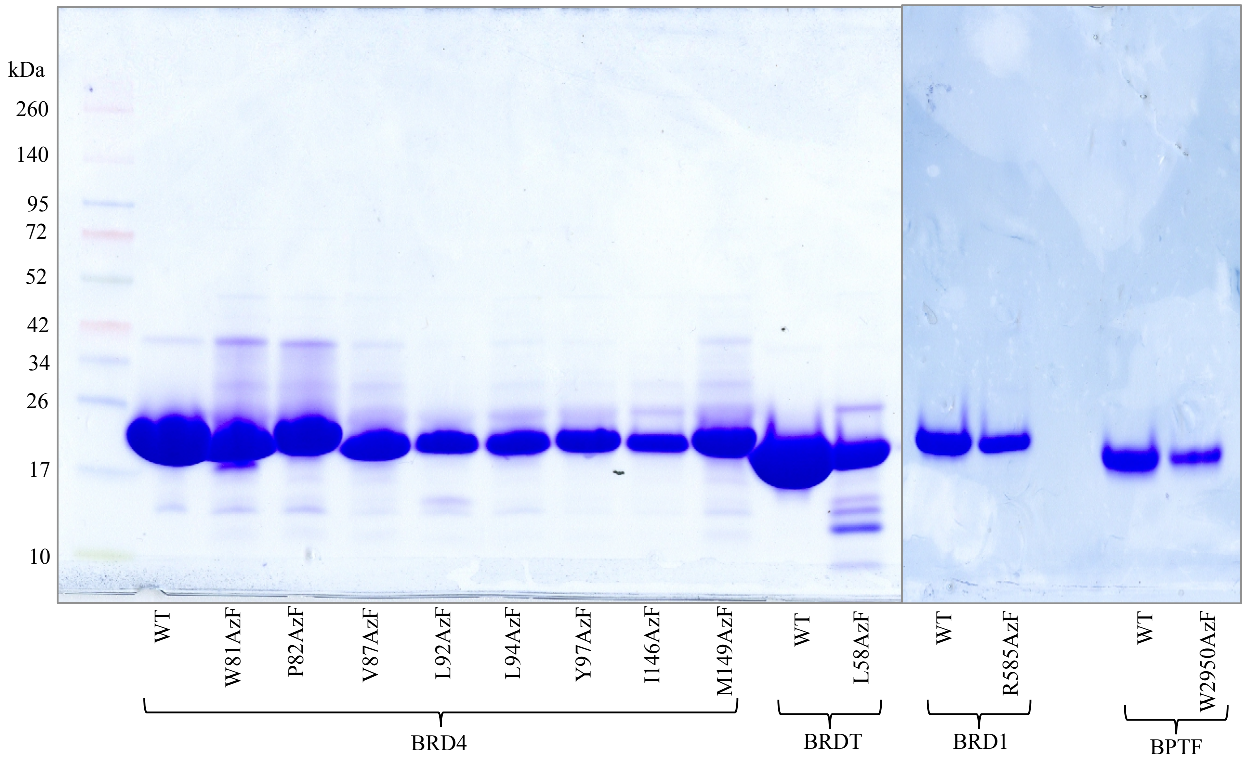

Supplementary Figure S1: Coomassie blue staining showing expression and purity of wild type BRD4-BD1, wild type BRDT-BD1, wild type BRD1, wild type BPTF and their pAzF containing mutants. LC-MS analyses of intact proteins confirmed the integrity of these proteins (See Supplementary Table S1).

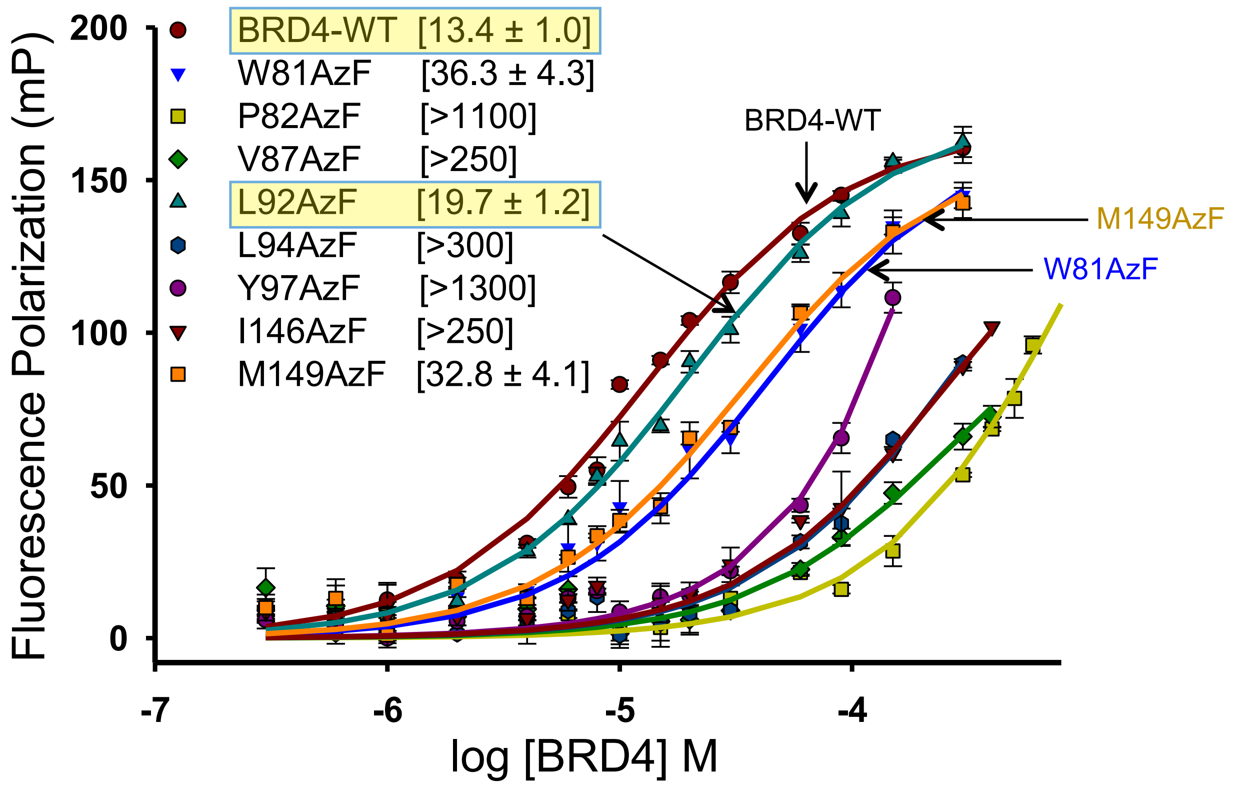

Supplementary Figure S2: Dissociation constants (K_d in μM as shown in parentheses) of BRD4 and its *p*AzF mutants towards the TAMRA-labeled tetra-acetylated histone peptide **2** as determined by background-corrected fluorescence polarization values. BRD4-WT and its L92AzF mutant show comparable binding towards the peptide. The data were fitted to a single-site binding equation $Y = B_{max} X / (K_d + X)$, where Y is the specific binding, B_{max} is the maximal binding and X is the concentration of the ligand, using the SigmaPlot software.[2,3] Error bars represent standard deviation from three independent measurements. We did not observe saturation in mP values for the weakly bound mutants (P82AzF, V87AzF, L94AzF, Y97AzF and I146AzF) even at higher concentrations. While the K_d values for V87AzF, L94AzF and I146AzF are estimated based on the approximate B_{max} values, the same for P82AzF and Y97AzF cannot be reliably estimated as the values are outside of the measurable range.

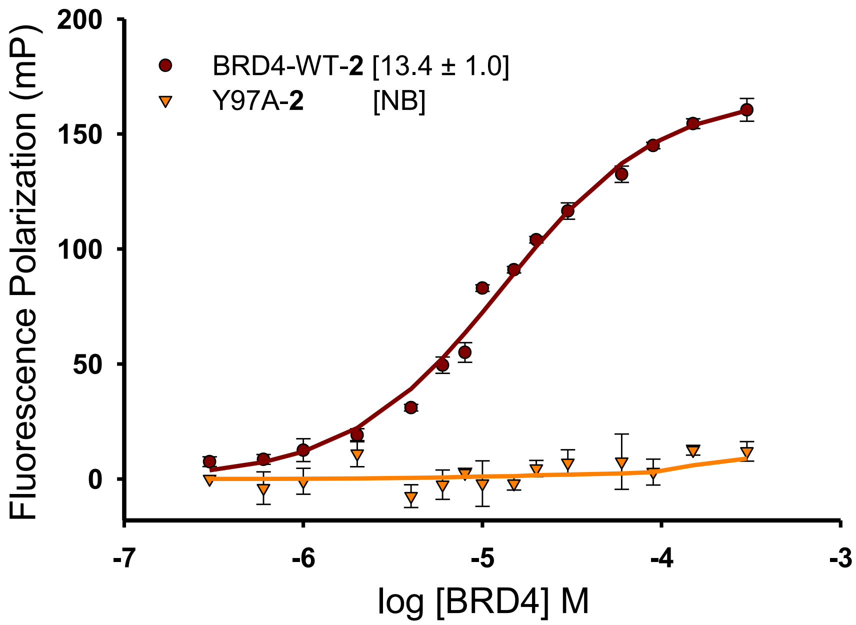

Supplementary Figure S3: Lack of binding of Y97A mutant to the TAMRA-attached tetra-acetylated histone peptide **2**. Error bars represent standard deviation from three independent measurements. Data analysis was performed similar to Figure S2. (NB= No binding)

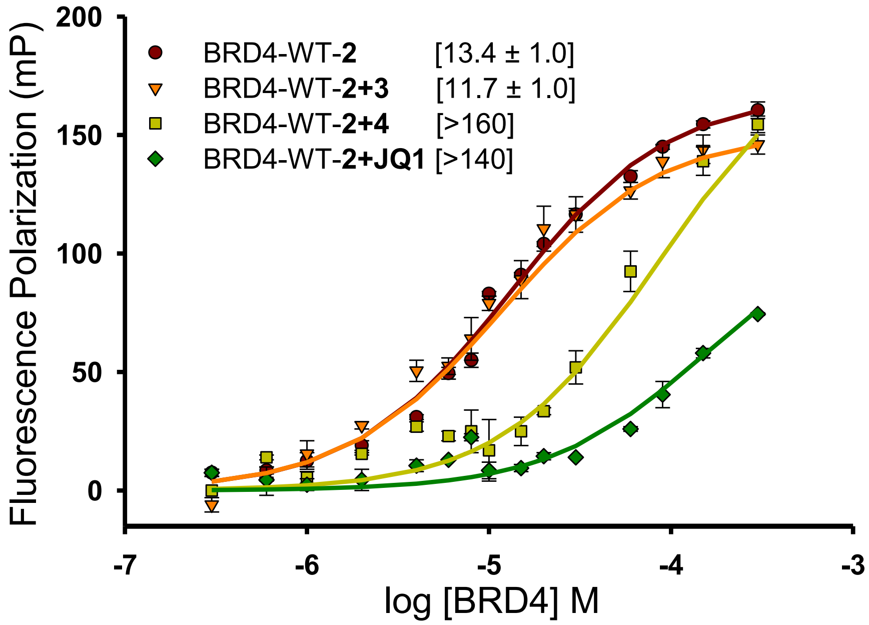

Supplementary Figure S4: Inhibitory effect of peptides **3** and **4**, and JQ1 on binding of BRD4-BD1 to **2**. While unlabeled tetra-acetylated peptide **4** and JQ1 substantially reduced the binding, peptide **3,** which bears no acetyl group, had no effect on the binding. Values in parentheses represent dissociation constant (K_d, μM). Error bars represent standard deviation from three independent measurements. Data analysis was performed similar to Figure S2.

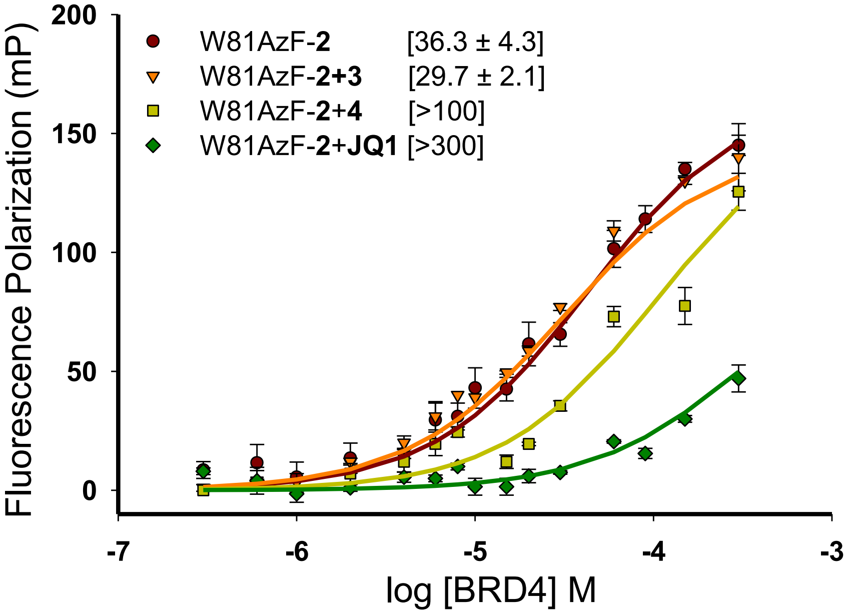

Supplementary Figure S5: Inhibitory effect of peptides **3** and **4**, and JQ1 on W81AzF binding to **2**. While unlabeled tetra-cetylated peptide **4** and JQ1 substantially reduced the binding, peptide **3,** which bears no acetyl group, had no effect on the binding. Values in parentheses represent dissociation constant (K_d, μM). Error bars represent standard deviation from three independent measurements. Data analysis was performed similar to Figure S2.

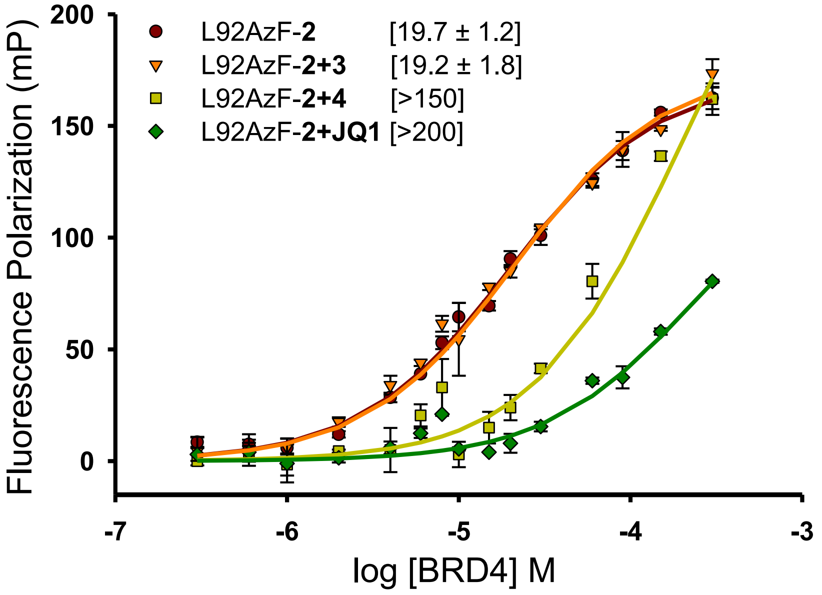

Supplementary Figure S6: Inhibitory effect of peptides **3** and **4**, and JQ1 on L92AzF binding to **2**. While unlabeled tetra-cetylated peptide **4** and JQ1 substantially reduced the binding, peptide **3,** which bears no acetyl group, had no effect on the binding. Values in parentheses represent

dissociation constant (K_d in μM). Error bars represent standard deviation from three independent measurements. Data analysis was performed similar to Figure S2.

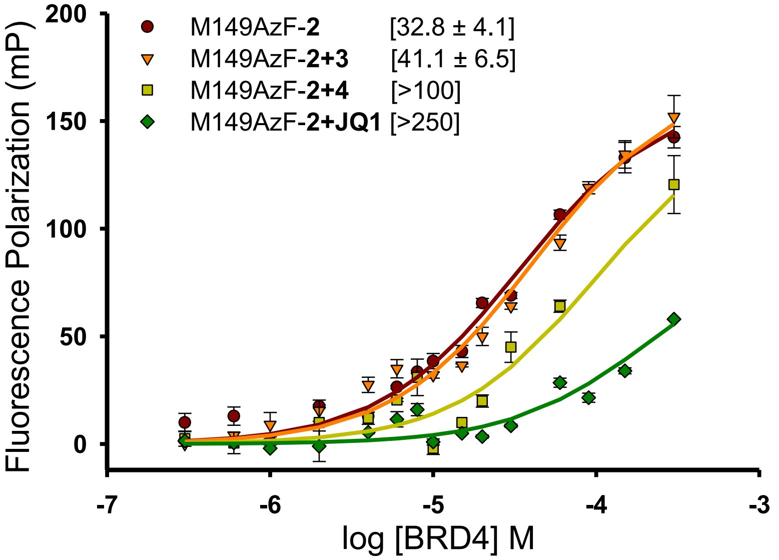

Supplementary Figure S7: Inhibitory effect of peptides **3** and **4**, and JQ1 on M149AzF binding to **2**. While unlabeled tetra-cetylated peptide **4** and JQ1 substantially reduced the binding, peptide **3**, which bears no acetyl group, had no effect on the binding. Values in parentheses represent dissociation constant (K_d in μM). Error bars represent standard deviation from three independent measurements. Data analysis was performed similar to Figure S2.

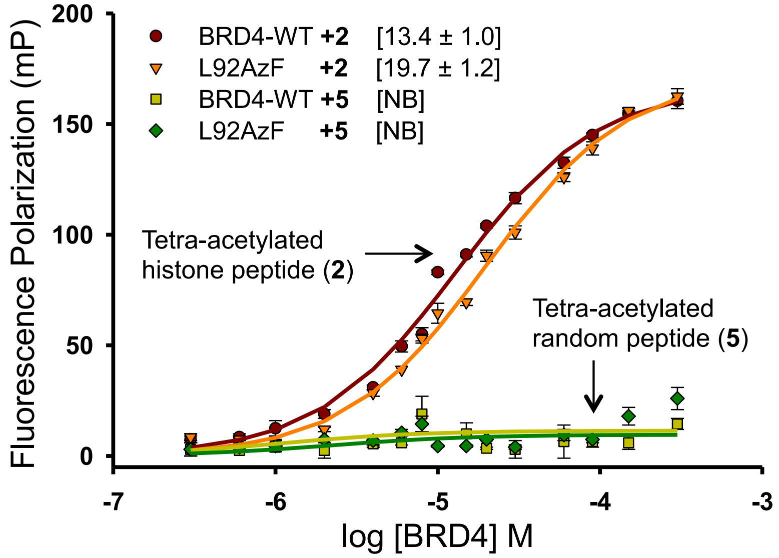

Supplementary Figure S8: Wild type BRD4-BD1 and its mutant L92AzF bind TAMRA- attached tetra-acetylated histone peptide **2**, but failed to bind the TAMRA-attached tetra-acetylated random peptide **5** [TAMRA-GGGGDNPQYK(Ac)YFK(Ac)APGK(Ac)VWLK(Ac)PCLI] although it

contains four acetyl groups. Values in parentheses represent dissociation constant (K_d in μM). Error bars represent standard deviation from three independent measurements. Data analysis was performed similar to Figure S2. (NB = No binding)

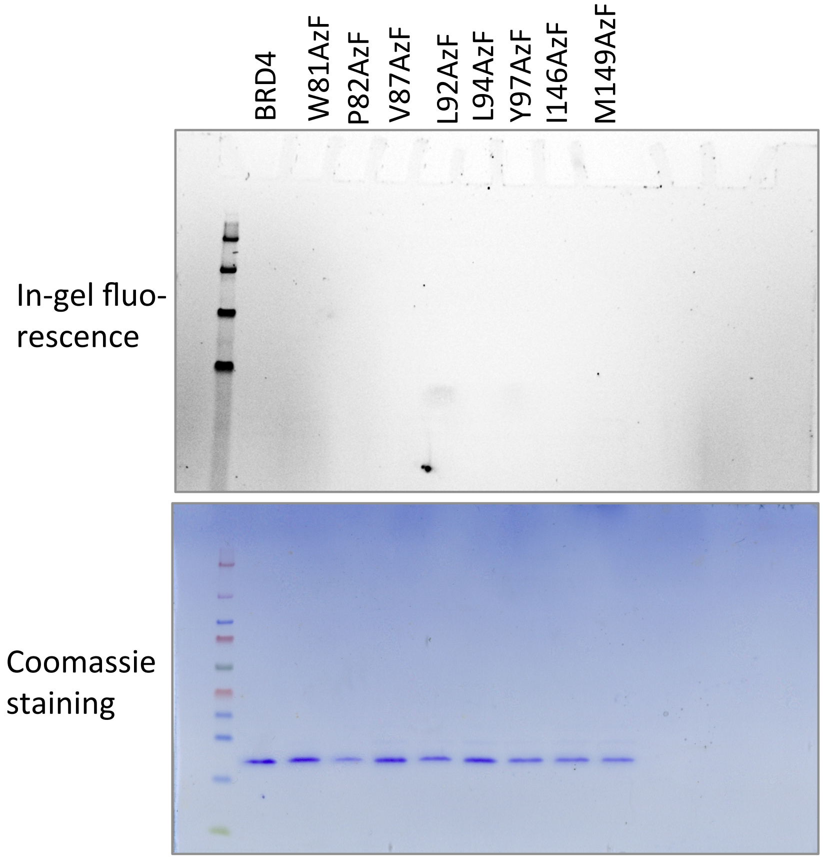

Supplementary Figure S9: Negative control experiment for crosslinking and in-gel fluorescence for BRD4 and its mutants towards peptide **2**. The proteins were incubated with the peptide **2** but were not exposed to UV light. Subsequent pull-down with Ni-NTA bead, washing and in-gel fluorescence did not show any fluorescently labeled band. Coomassie staining of the same gel clearly indicated the presence of proteins in all the samples.

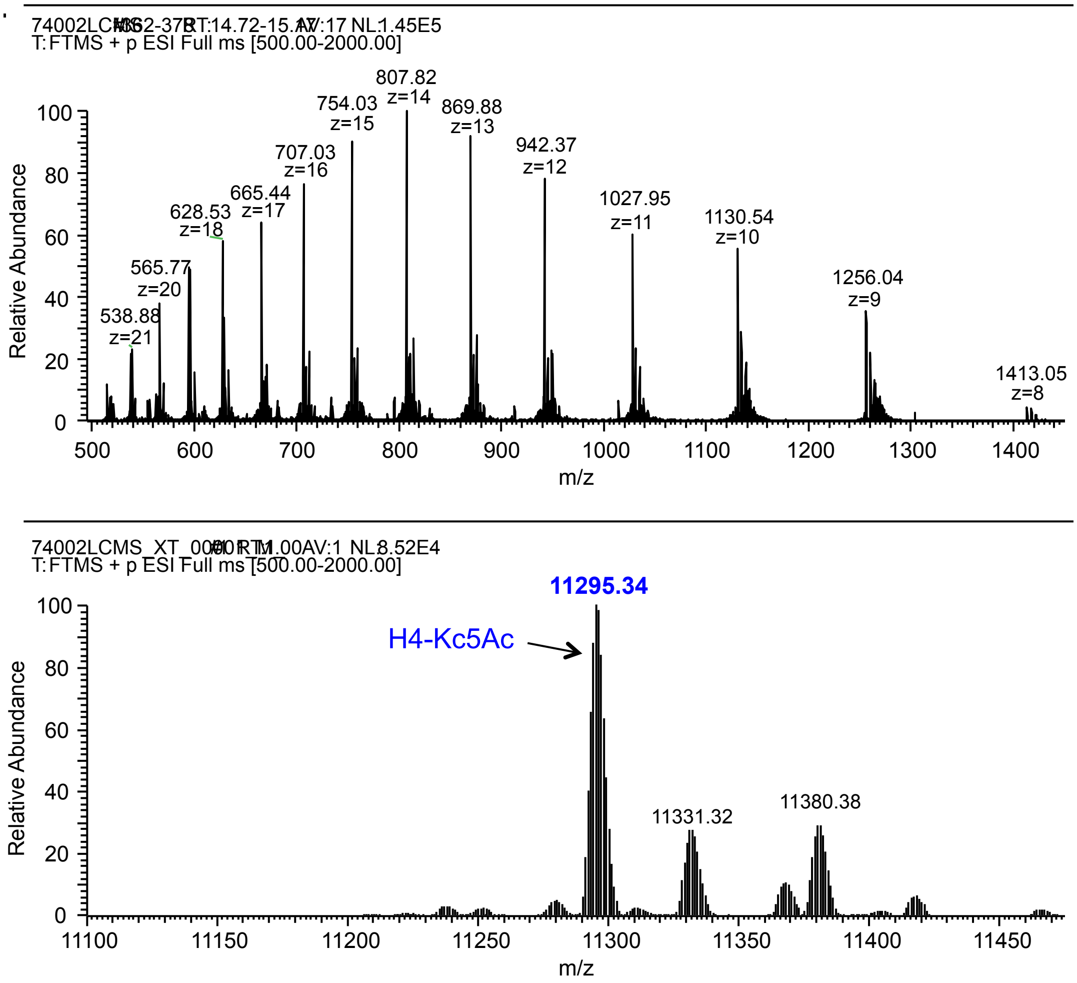

Supplementary Figure S10: ESI LC-MS spectra of histone H4 Kc5Ac. The major peak at 11295.34 corresponds to the expected molecular weight of H4-Kc5Ac. Proteins corresponding to minor peaks observed in mass spectrum were not separable in HPLC.

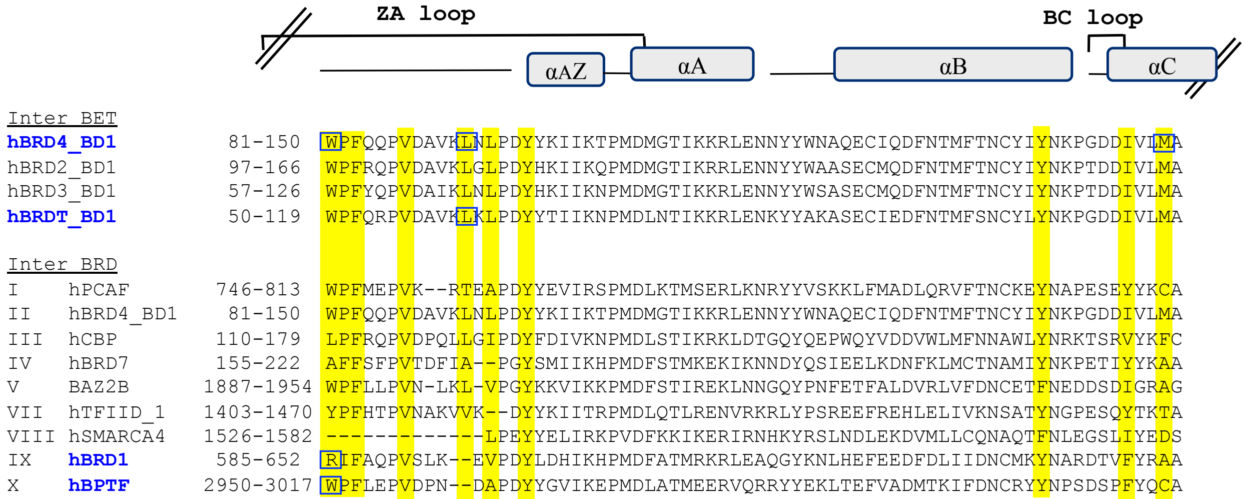

Supplementary Figure S11: Sequence alignment of BET (bromodomain and extra terminal) bromodomains (BRD2, BRD3, BRD4 and BRDT) and non-BET bromodomains (PCAF, CBP, BRD7, BAZ2B, TFIID, SMARCA4, BRD1 and BPTF).[2] Highlighted conserved residues form aromatic cage in these bromodomain proteins to recognize acetylated lysines. To demonstrate generality, L61 of BRDT (equivalent to L92 of BRD4) and R585 of BRD1 and W2950 of BPTF (equivalent to W81 of BRD4) are selected to introduce pAzF amino acid as shown in the blue boxes.

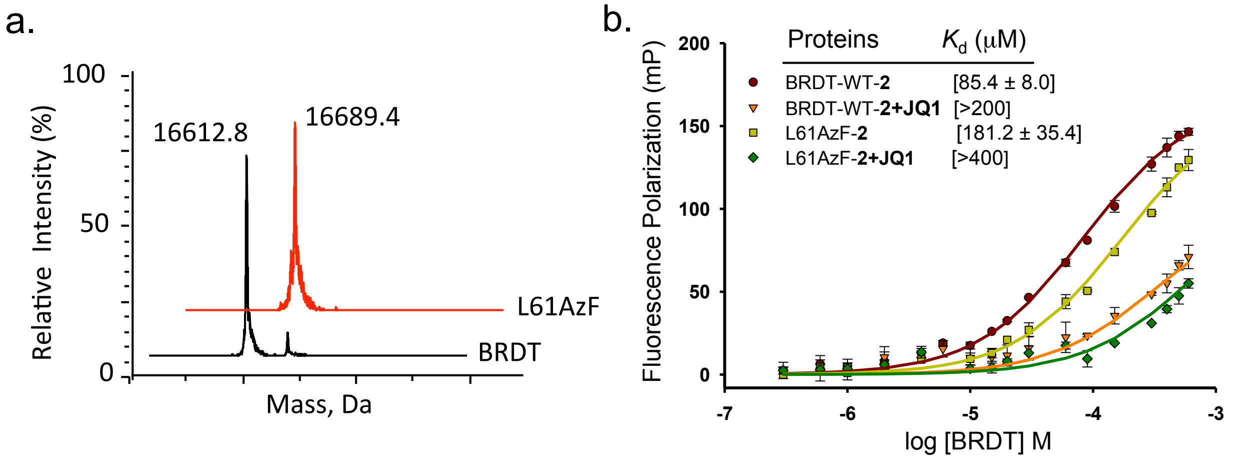

Supplementary Figure S12: (a) LC-MS spectra of wild type BRDT and its L61AzF. (b) Dissociation constants (K_d in μM) of BRDT and its L61AzF mutant towards the TAMRA-attached tetra-acetylated histone peptide **2** as measured by fluorescence polarization. JQ1 efficiently inhibited binding of these proteins towards **2**. Error bars represent standard deviation from three independent measurements. Data analysis was performed similar to Figure S2.

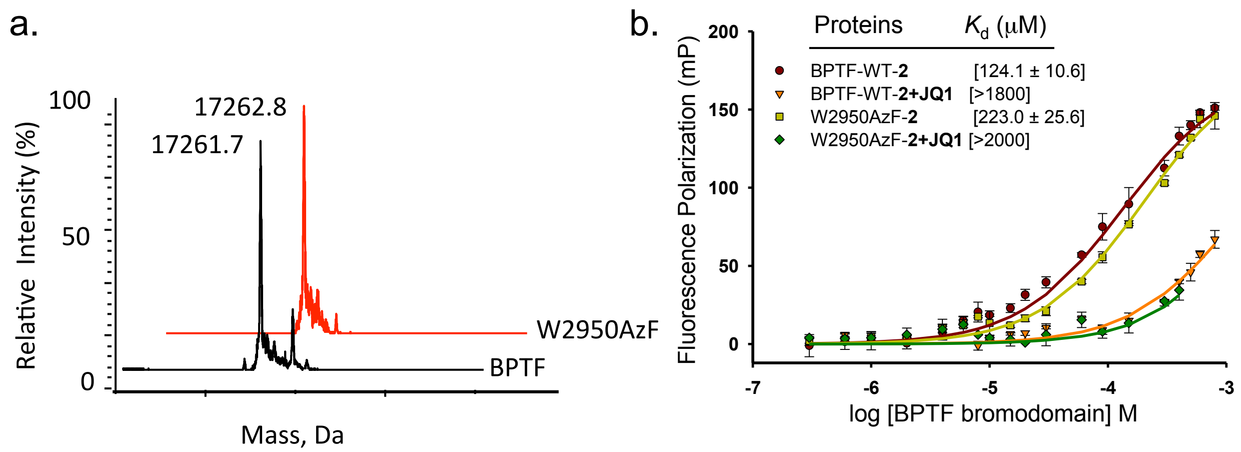

Supplementary Figure S13: (a) LC-MS spectra of wild type BPTF and its W2950AzF. (b) Dissociation constants (K_d in μM) of BPTF and its W91AzF mutant towards the TAMRA-attached tetra-acetylated histone peptide **2** as measured by fluorescence polarization. JQ1 efficiently inhibited binding of these proteins towards **2**. Error bars represent standard deviation from three independent measurements. Data analysis was performed similar to Figure S2.

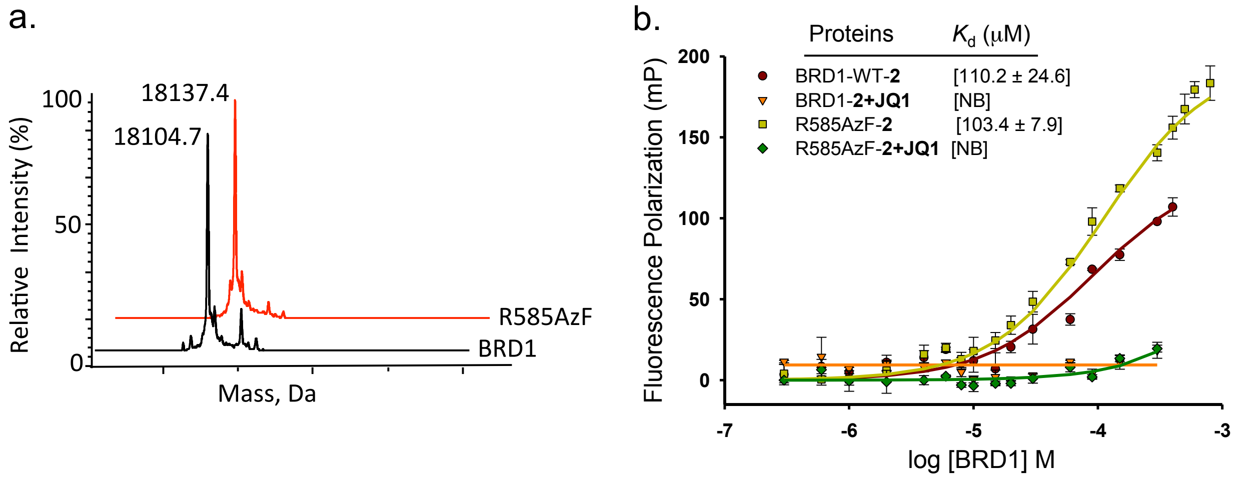

Supplementary Figure S14: (a) LC-MS spectra of wild type BRD1 and its R595AzF. (b) Dissociation constants (K_d in μM) of BRD1 and its W2950AzF mutant towards the TAMRA-attached tetra-acetylated histone peptide **2** as measured by fluorescence polarization. Error bars represent standard deviation from three independent measurements. Data analysis was performed similar to Figure S2. Data analysis was performed similar to Figure S2. (NB = no binding)

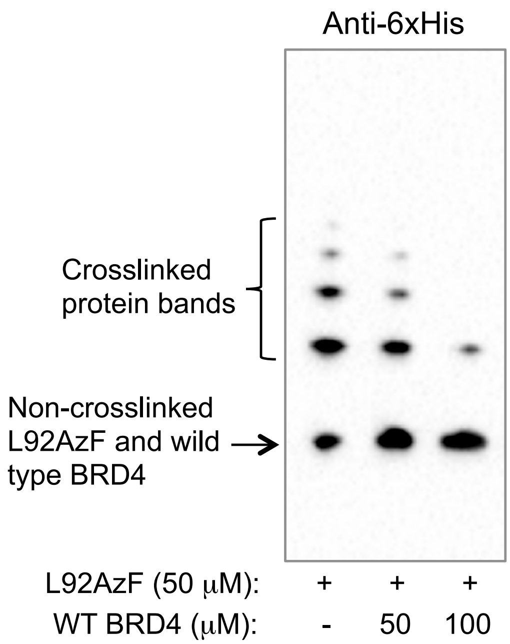

Supplementary Figure S15: Addition of increasing amount of wild type BRD4 (0 – 100 μM) in the crosslinking experiment leads to decrease in crosslinking of the L92AzF mutant (50 μM) towards the interacting partners present in HEK293T cell lysates.

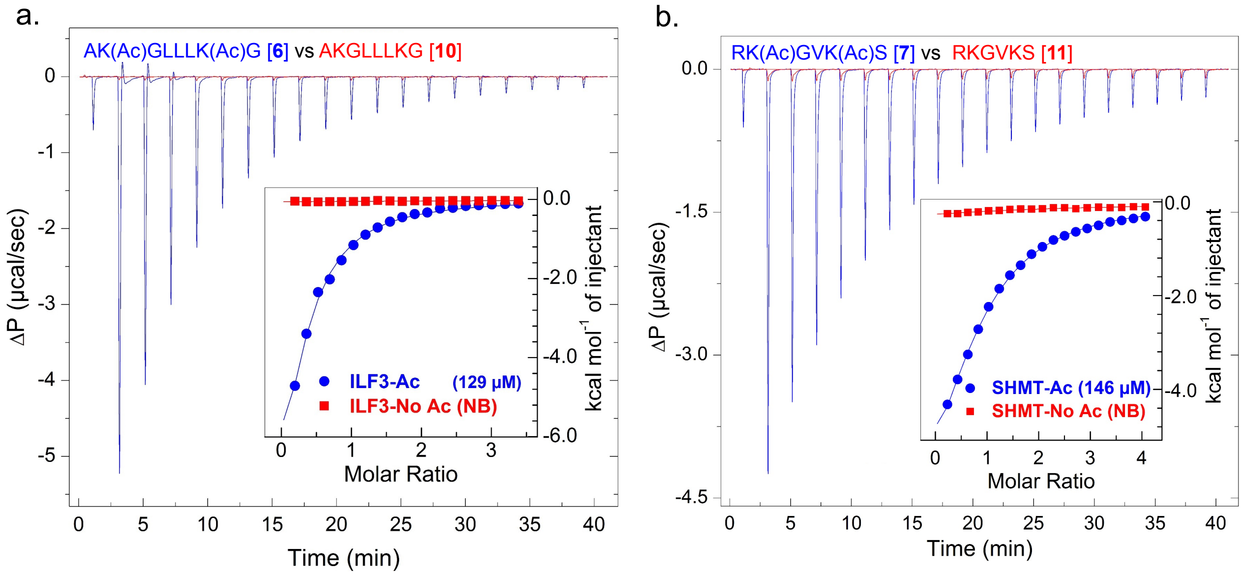

Supplementary Figure S16: Binding of acetylated and non-acetylated ILF3 (a) and SHMT (b) peptides to wild type BRD4 bromodomain as measured by isothermal titration calorimetry. Although the acetylated peptides showed strong binding (K_d in μM), their corresponding non-acetylated counterpart failed completely to bind BRD4. (NB = no binding)

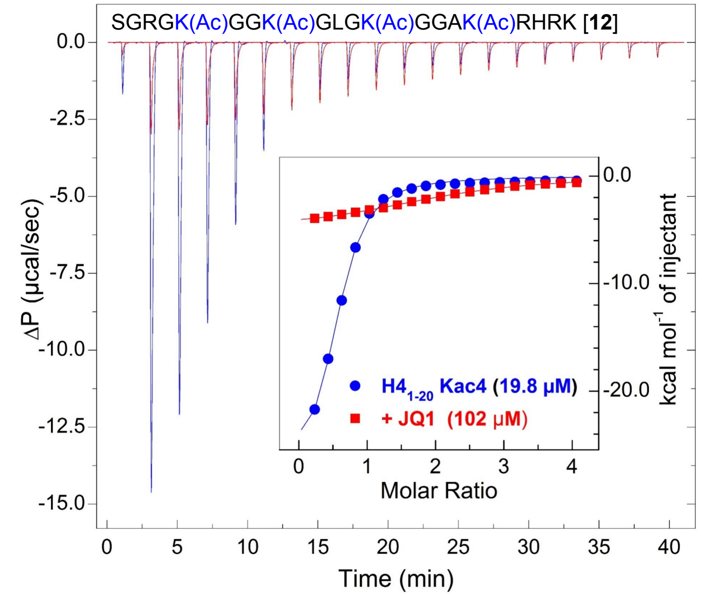

Supplementary Figure S17: Binding of tetracaetylated histone H4 peptide **12** to wild type BRD4 as determined by isothermal titration calorimetry (K_d in μM). JQ1 significantly inhibited the binding. The K_d values obtained through ITC experiments corroborated well to the data obtained from fluorescence polarization-based binding experiments (Figures S2 and S4).

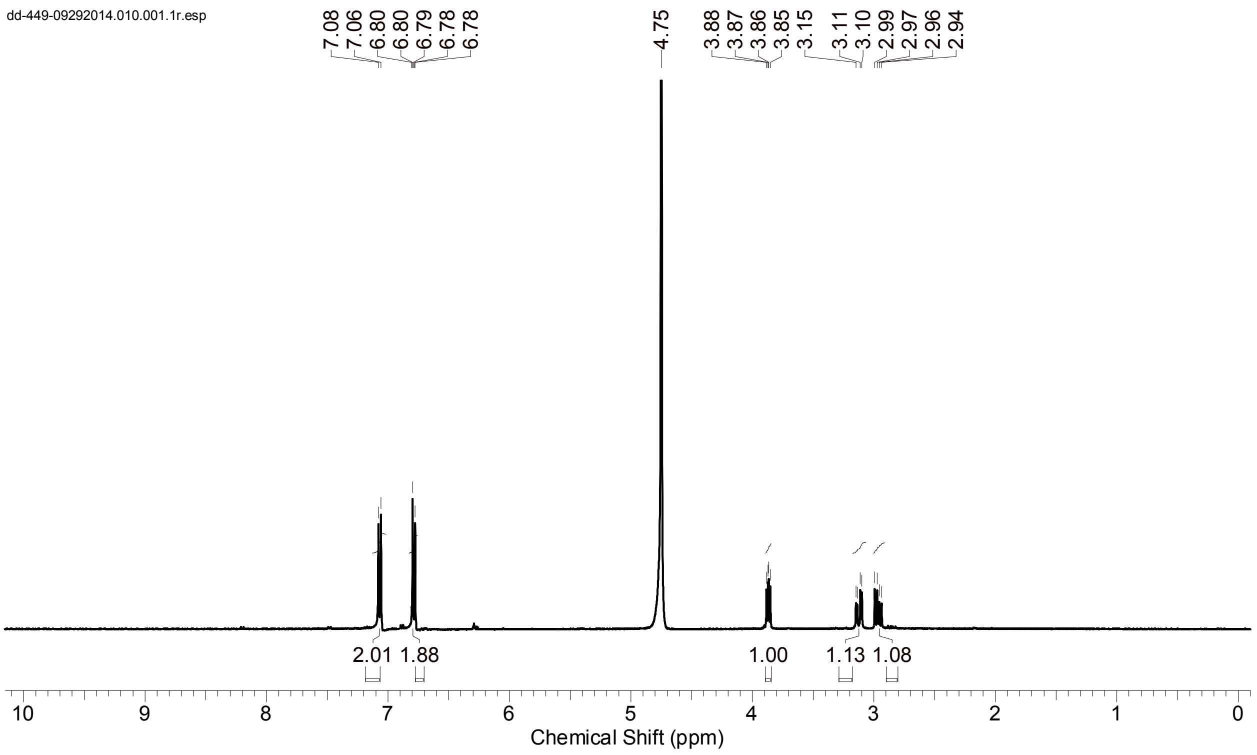

Supplementary Figure 18: ^{1}H NMR spectrum of 4-amino phenylalanine

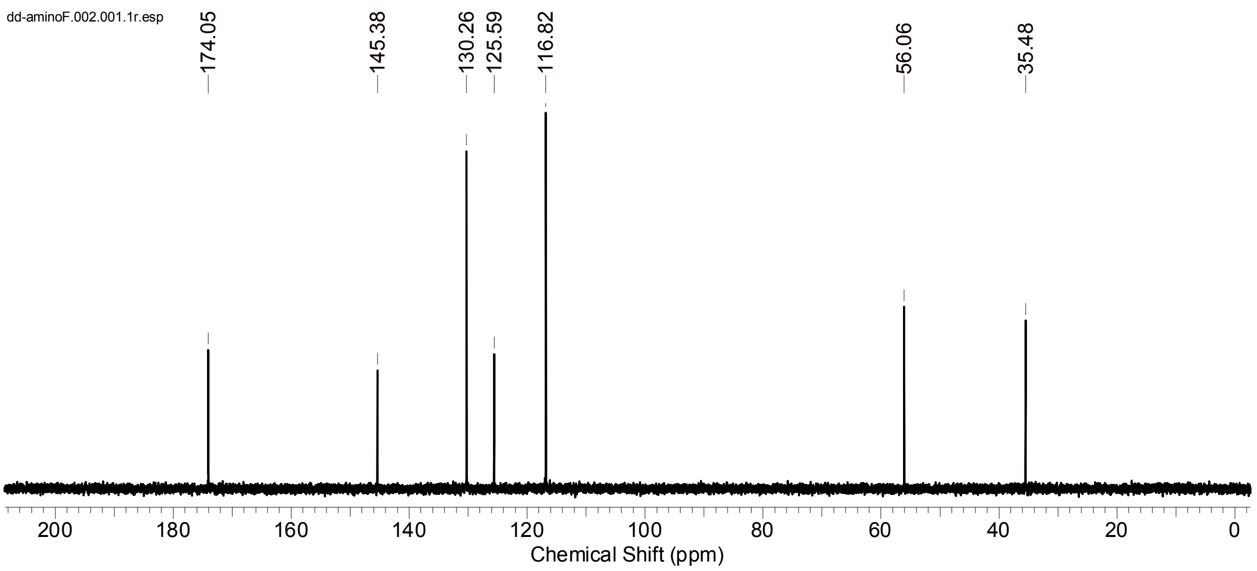

Supplementary Figure 19: ^{13}C NMR spectrum of 4-amino phenylalanine

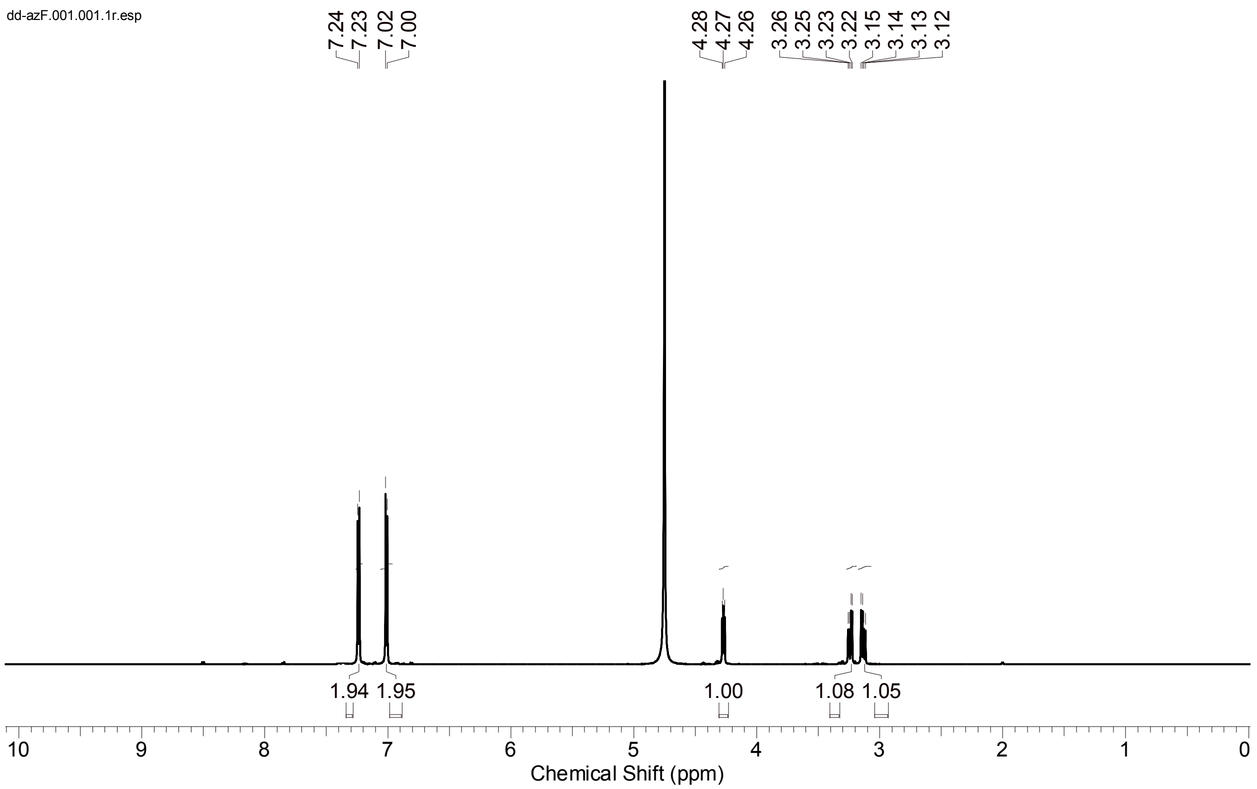

Supplementary Figure 20: ^{1}H NMR spectrum of 4-azido phenylalanine (*p*AzF) **1**

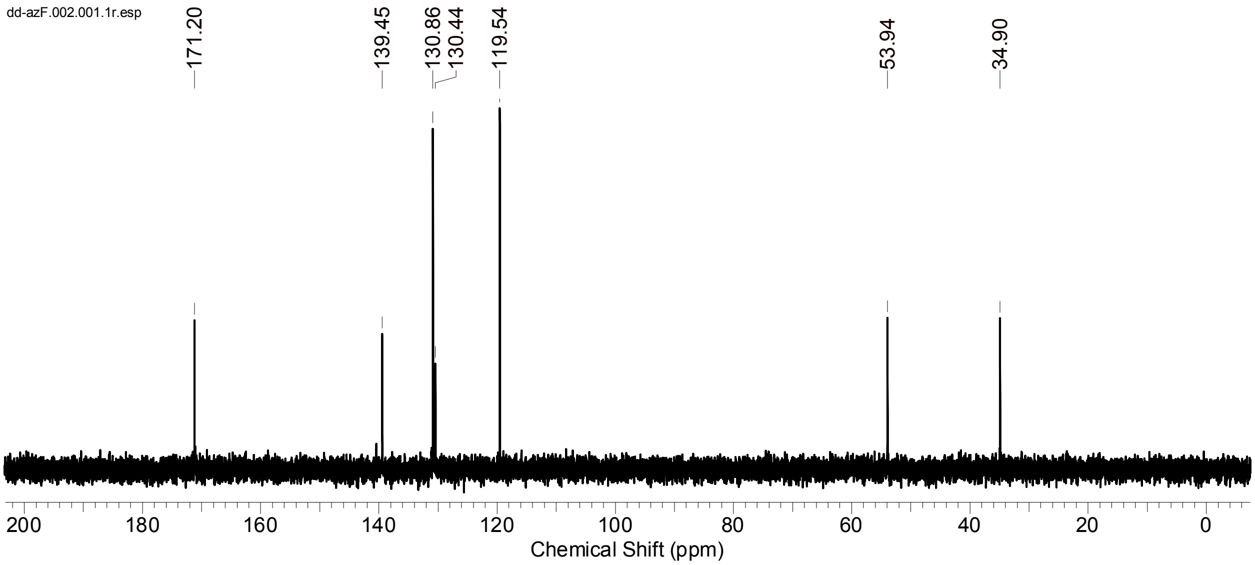

Supplementary Figure 21: ^{13}C NMR spectrum of 4-azido phenylalanine (*p*AzF) **1**

Protein name	Amino acid Mol.wt	NAA Mol.wt AzF	Modification	Mole. wt difference from WT	Calculated Mol.wt	Observed (LC-MS)
**BRD4-WT**	-	-	-	-	17549.07	17548.7
**W81AzF**	Trp-204.23	206.20	W81 to AzF	1.97	17551.04	17550.2
**P82AzF**	Pro-115.13	206.20	P82 to AzF	91.07	17640.14	17639.8
**V87AzF**	Val-117.15	206.20	V87 to AzF	89.05	17638.12	17637.3
**L92AzF**	Leu-131.17	206.20	L92 to AzF	75.03	17624.1	17623.3
**L94AzF**	Leu-131.17	206.20	L94 to AzF	75.03	17624.1	17623.0
**Y97AzF**	Tyr-181.19	206.20	Y97 to AzF	25.01	17574.08	17573.7
**I146AzF**	Ile-131.17	206.20	I146 to AzF	75.03	17624.1	17624.3
**M149AzF**	Met-149.21	206.20	M149 to AzF	56.99	17606.06	17605.6
**BRDT-WT**	-	-	-	-	16614.09	16612.8
**L61AzF**	Leu-131.17	206.20	L61 to AzF	75.03	16689.12	16689.4
**BPTF-WT**	-	-	-	-	17262.47	17261.7
**W2950AzF**	Trp-204.23	206.20	W2950 to AzF	1.97	17264.44	17262.8
**BRD1-WT**	-	-	-	-	18105.58	18104.7
**R585AzF**	Arg-174.20	206.20	R585 to AzF	32	18137.58	18137.4

Supplementary Table S1: Calculated and experimental molecular weights of wild type BRD4-BD1, BRDT-BD1, BPTF bromodomain, BRD1 bromodomain and their *p*AzF mutants.

Supplementary Table S2 contains proteomic data and has been uploaded as a separate Excel file.

Protein	Peptide	Peptide sequence	Protein (µM)	Peptide (mM)	K_D (µM)	N	ΔH (kcal/mol)	TΔS (kcal/mol)	ΔG (kcal/mol)
BRD4-BD1	ILF3	AK(Ac)GLLLK(Ac)G (**6**)	300	5	129.8 ± 15.0	0.28 ± 0.047	-14.47	-9.33	-5.14
	SHMT	RK(Ac)GVK(Ac)S (**7**)	200	4	146.4 ± 6.0	0.62 ± 0.078	-13.12	-8.24	-4.88
	HNRNPK	GK(Ac)GGK(Ac)NIK(Ac)A (**8**)	200	4	234.2 ± 23.5	0.61 ± 0.093	-24.31	-19.50	-4.81
	PDIA1	AK(Ac)AAGK(Ac)LK(Ac)A (**9**)	250	5	353.3 ± 7.3	0.57 ± 0.023	-19.57	-15.01	-4.56
	ILF3-No Ac	AKGLLLKG (**10**)	300	5	NB				
	SHMT-No Ac	RKGVKS (**11**)	200	4	NB				
	H4K5/8/12/16	SGRGK(Ac)GGK(Ac)GLGK(Ac)GGAK(Ac)RHRK (**12**)	200	4	19.8 ± 1.8	0.54 ± 0.012	-28.21	-22.01	-6.20

Supplementary Table S3. Thermodynamic parameters measured by isothermal titration calorimetry (ITC) for the binding of above peptides **6**-**12** to BRD4-BD1. Conditions are detailed in the experimental section.

Protein	Peptide	Peptide sequence	Protein (µM)	JQ1 (µM)	Peptide (mM)	K_D (µM)	N	ΔH (kcal/mol)	TΔS (kcal/mol)	ΔG (kcal/mol)
BRD4-BD1	ILF3	AK(Ac)GLLLK(Ac)G (**6**)	300	2	5	365.9 ± 68.3	0.87 ± 0.323	-2.86	-1.67	-1.19
	SHMT	RK(Ac)GVK(Ac)S (**7**)	200	1	4	395.2 ± 45.2	0.75 ± 0.155	-6.77	-2.28	-4.49
	HNRNPK	GK(Ac)GGK(Ac)NIK(Ac)A (**8**)	200	2	4	416.6 ± 23.5	1.42 ± 0.069	-4.73	-0.27	-4.46
	PDIA1	AK(Ac)AAGK(Ac)LK(Ac)A (**9**)	250	1	5	495.2 ± 15.3	0.56 ± 0.043	-1.38	-0.28	-1.10
	H4K5/8/12/16	SGRGK(Ac)GGK(Ac)GLGK(Ac)GGAK(Ac)RHRK (**12**)	200	2	4	102.3 ± 2.6	2.06 ± 0.010	-5.03	-0.22	-4.81

Supplementary Table S4. Thermodynamic parameters measured by isothermal titration calorimetry (ITC) for the binding of above peptides **6**-**9**,**12** to BRD4-BD1 in presence of inhibitor JQ1. Conditions are detailed in the experimental section.

GENE	VECTOR	AFFINITY TAG	RESISTANCE
BRD4	pNIC28-Bsa4	N-6xHis	Kanamycin
BRDT	pNIC28-Bsa4	N-6xHis	Kanamycin
BPTF	pNIC28-Bsa4	N-6xHis	Kanamycin
BRD1	pNIC28-Bsa4	N-6xHis	Kanamycin
HISTONE H4	pDEST	None	Ampicillin

Supplementary Table S5. List of the genes used in the current study. The expression vector, antibiotic resistance and the affinity tag present for protein purification are provided.

MUTATION	FORWARD PRIMERS
BRD4-W81TAG	5'-CACCAGTTTGCATAGCCTTTCCAGCAGCC-3'
BRD4-P82TAG	5'-CACCAGTTTGCATGGTAGTTCCAGCAGCCTGTG-3'
BRD4-F83TAG	5'-GCATGGCCTTAGCAGCAGCCTGTG-3'
BRD4-V87TAG	5'-CCTTTCCAGCAGCCTTAGGATGCCGTCAAGCTG-3'
BRD4-L92TAG	5'-GTGGATGCCGTCAAGTAGAACCTCCCTGAT-3'
BRD4-L94TAG	5'-GCCGTCAAGCTGAACTAGCCTGATTACTATAAG-3'
BRD4-Y97TAG	5'-GCTGAACCTCCCTGATTAGTATAAGATCA-3'
BRD4-C136TAG	5'-CACTATGTTTACAAATTAGTACATCTACAAC-3'
BRD4-Y139TAG	5'-CAAATTGTTACATCTAGAACAAGCCTGGAGATGAC-3'
BRD4-D144TAG	5'-CAACAAGCCTGGATAGGACATAGTCTT-3'
BRD4-D145TAG	5'-CAAGCCTGGAGATTAGATAGTCTTAATGGC-3'
BRD4-I146TAG	5'-GCCTGGAGATGACTAGGTCTTAATGGC-3'
BRD4-M149TAG	5'-GATGACATAGTCTTATAGGCAGAAGCTCTGGAA-3'
BRD4-Y97A	5'-GCTGAACCTCCCTGATGCCTATAAGATCA-3'
BRDT-L61TAG	5'-GTGGATGCTGTGAAATAGCAGTTGCCTGAT-3'
BPTF-W2950TAG	5'-GCGCATAAAATGGCGTAGCCGTTTCTGGAACCG-3'
BRD1-R585TAG	5'-GACAAGGACCCCGCCTAGATATTTGCGCAGCCCG-3'
H4-K5C	5'-GTCTGGTCGTGGTTGCGGTGGTAAAGGT-3'

Supplementary Table S6. List of primers designed for site-directed mutagenesis. Reverse primers used are the reverse-complement to the given forward primers.

PROTEINS	BACTERIAL CELLS FOR EXPRESSION
BRD4-WT	BL21 codon plus [DE3] RIPL
BRD4-W81AzF	BL21 [DE3] Star
BRD4-P82AzF	BL21 [DE3] Star
BRD4-F83AzF	BL21 [DE3] Star
BRD4-V87AzF	BL21 [DE3] Star
BRD4-L92AzF	BL21 [DE3] Star
BRD4-L94AzF	BL21 [DE3] Star
BRD4-Y97AzF	BL21 [DE3] Star
BRD4-C136AzF	BL21 [DE3] Star
BRD4-Y139AzF	BL21 [DE3] Star
BRD4-D144AzF	BL21 [DE3] Star
BRD4-D145AzF	BL21 [DE3] Star
BRD4-I146AzF	BL21 [DE3] Star
BRD4-M149AzF	BL21 [DE3] Star
BRD4-L92A	BL21 codon plus [DE3] RIPL
BRD4-L92G	BL21 codon plus [DE3] RIPL
BRD4-L92Y	BL21 codon plus [DE3] RIPL
BRD4-Y97A	BL21 codon plus [DE3] RIPL
BRDT-WT	BL21 codon plus [DE3] RIPL
BRDT-L61AzF	BL21 [DE3] Star
BPTF-WT	BL21 codon plus [DE3] RIPL
BPTF-W2950AzF	BL21 [DE3] Star
BRD1-WT	BL21 codon plus [DE3] RIPL
BRD1-R585AzF	BL21 [DE3] Star
Histone-H4-WT	BL21 codon plus [DE3] RIPL
H4-K5C	BL21 codon plus [DE3] RIPL

Supplementary Table S7. List of competent bacterial cells used in the current study for the expression of indicated proteins.

17. References

1. T. Wieland, A. von Dungen and C. Birr, *Justus Liebigs Ann. Chem.*, 1971, **752**, 109-114.
2. J. W. Chin, S. W. Santoro, A. B. Martin, D. S. King, L. Wang and P. G. Schultz, *J. Am. Chem. Soc.*, 2002, **124**, 9026-9027.
3. M. Jung, M. Philpott, S. Muller, J. Schulze, V. Badock, U. Eberspacher, D. Moosmayer, B. Bader, N. Schmees, A. Fernandez-Montalvan and B. Haendler, *J. Biol. Chem.*, 2014, **289**, 9304-9319.

4. A. Dhall, S. Wei, B. Fierz, C. L. Woodcock, T. H. Lee and C. Chatterjee, *J. Biol. Chem.*, 2014, **289**, 33827-33837.
5. P. Drogaris, V. Villeneuve, C. Pomies, E. H. Lee, V. Bourdeau, E. Bonneil, G. Ferbeyre, A. Verreault and P. Thibault, *Sci. Rep.*, 2012, **2**, 220.
6. A. Shevchenko, H. Tomas, J. Havlis, J. V. Olsen and M. Mann, *Nat. Protoc.*, 2006, **1**, 2856-2860.
